# Wastewater monitoring for detection of public health markers during the COVID-19 pandemic: Near-source monitoring of schools in England over an academic year

**DOI:** 10.1371/journal.pone.0286259

**Published:** 2023-05-30

**Authors:** Francis Hassard, Milan Vu, Shadi Rahimzadeh, Victor Castro-Gutierrez, Isobel Stanton, Beata Burczynska, Dirk Wildeboer, Gianluca Baio, Mathew R. Brown, Hemda Garelick, Jan Hofman, Barbara Kasprzyk-Hordern, Azeem Majeed, Sally Priest, Hubert Denise, Mohammad Khalifa, Irene Bassano, Matthew J. Wade, Jasmine Grimsley, Lian Lundy, Andrew C. Singer, Mariachiara Di Cesare

**Affiliations:** 1 Cranfield University, Bedfordshire, United Kingdom; 2 Institute for Nanotechnology and Water Sustainability, University of South Africa, Johannesburg, South Africa; 3 Department of Natural Science, School of Science and Technology, Middlesex University, London, United Kingdom; 4 Environmental Pollution Research Centre (CICA), Universidad de Costa Rica, Montes de Oca, Costa Rica; 5 UK Centre for Ecology and Hydrology, Wallingford, United Kingdom; 6 Department of Statistical Science, University College London, London, United Kingdom; 7 School of Engineering, Newcastle University, Newcastle-upon-Tyne, United Kingdom; 8 Environmental Monitoring for Health Protection, UK Health Security Agency, London, United Kingdom; 9 Water Innovation & Research Centre, Department of Chemical Engineering, University of Bath, Bath, United Kingdom; 10 Water Innovation & Research Centre, Department of Chemistry, University of Bath, Bath, United Kingdom; 11 Department of Primary Care & Public Health, Imperial College Faculty of Medicine, London, United Kingdom; 12 Institute of Public Health and Wellbeing, University of Essex, Colchester, United Kingdom; University of Helsinki: Helsingin Yliopisto, FINLAND

## Abstract

**Background:**

Schools are high-risk settings for infectious disease transmission. Wastewater monitoring for infectious diseases has been used to identify and mitigate outbreaks in many near-source settings during the COVID-19 pandemic, including universities and hospitals but less is known about the technology when applied for school health protection. This study aimed to implement a wastewater surveillance system to detect SARS-CoV-2 and other public health markers from wastewater in schools in England.

**Methods:**

A total of 855 wastewater samples were collected from 16 schools (10 primary, 5 secondary and 1 post-16 and further education) over 10 months of school term time. Wastewater was analysed for SARS-CoV-2 genomic copies of N1 and E genes by RT-qPCR. A subset of wastewater samples was sent for genomic sequencing, enabling determination of the presence of SARS-CoV-2 and emergence of variant(s) contributing to COVID-19 infections within schools. In total, >280 microbial pathogens and >1200 AMR genes were screened using RT-qPCR and metagenomics to consider the utility of these additional targets to further inform on health threats within the schools.

**Results:**

We report on wastewater-based surveillance for COVID-19 within English primary, secondary and further education schools over a full academic year (October 2020 to July 2021). The highest positivity rate (80.4%) was observed in the week commencing 30^th^ November 2020 during the emergence of the Alpha variant, indicating most schools contained people who were shedding the virus. There was high SARS-CoV-2 amplicon concentration (up to 9.2x10^6^ GC/L) detected over the summer term (8th June - 6th July 2021) during Delta variant prevalence. The summer increase of SARS-CoV-2 in school wastewater was reflected in age-specific clinical COVID-19 cases. Alpha variant and Delta variant were identified in the wastewater by sequencing of samples collected from December to March and June to July, respectively. Lead/lag analysis between SARS-CoV-2 concentrations in school and WWTP data sets show a maximum correlation between the two-time series when school data are lagged by two weeks. Furthermore, wastewater sample enrichment coupled with metagenomic sequencing and rapid informatics enabled the detection of other clinically relevant viral and bacterial pathogens and AMR.

**Conclusions:**

Passive wastewater monitoring surveillance in schools can identify cases of COVID-19. Samples can be sequenced to monitor for emerging and current variants of concern at the resolution of school catchments. Wastewater based monitoring for SARS-CoV-2 is a useful tool for SARS-CoV-2 passive surveillance and could be applied for case identification and containment, and mitigation in schools and other congregate settings with high risks of transmission. Wastewater monitoring enables public health authorities to develop targeted prevention and education programmes for hygiene measures within undertested communities across a broad range of use cases.

## Introduction

Since the first detection of SARS-CoV-2 in wastewater in March 2020 [1], the potential for wastewater monitoring to support pandemic mitigation measures has received considerable international attention [2, 3]. Wastewater is an aggregate of domestic and industrial effluents and stormwater, containing a mix of faeces, urine, mucus, and vomit from the ‘upstream’ population, which can be interrogated for population health purposes [4]. Specifically, viruses, bacteria, fungi, protists, anthropogenic chemicals (pharmaceuticals, personal care products, detergents, fragrances etc.), and human metabolites are shed into a sewer network, offering the potential to support public health decision makers through their analysis over time and space.

It has been extensively reported that SARS-CoV-2 can be recovered in various bodily fluids such as saliva [5], sputum [6, 7], faeces [6, 8], and urine [8, 9]. Wastewater-based epidemiology proliferated during the COVID-19 pandemic because it offered a rapid, low-cost, non-invasive method for capturing symptomatic, pre-symptomatic, and asymptomatic cases at a community level, including those from hard-to-reach and/or reticent to test populations [10, 11]. Wastewater monitoring can generate less biased insights than clinical testing of individuals as it does not require consent to sample from a sewer system [12]. There is considerable debate about whether wastewater data is a leading indicator of clinical cases (i.e., temporally ahead of). Since wastewater can be immediately analysed from shedding individuals at a population level it can feasibly offer more rapid insights than clinical approaches, which require the infected individual to proactively be tested for infection, something that would normally occur days after the start of an infection. In addition, this approach is also biased toward those with health seeking behaviour, missing out on those reluctant to test. Evidence suggests the link between cases and wastewater data depends on epidemiological (including biological, and health systems factors of specific populations of interest) [13], as well as (infra) structural aspects (i.e. the scale, frequency of clinical testing and pipe network characteristics). Whilst both clinical and wastewater samples require laboratory processing (analyte extraction), wastewater-derived SARS-CoV-2 RNA typically occurs in a more degraded form and at lower titre in comparison to clinical samples. However, despite this challenge, extracted RNA is amenable to genomic sequencing, providing an opportunity to continuously monitor and map the occurrence and spread of new variants [14] and their sub-lineages [4].

Schools are known to provide a conducive environment for the transmission of both respiratory and enteric infections through large numbers of people mixing regularly and at close quarters, in potentially poorly ventilated settings [15–17]. School age students can also be individually vulnerable due to stress and poor hygiene practices [18]. The role of schools as amplifiers of COVID-19 infections was not well-established at the onset of the COVID-19 pandemic [19] due to the unclear role of children in disease prevalence or transmission [20, 21] and their systematic under-testing [15]. As data emerged on school-age children, there was often close coupling between community cases and infections in children, suggesting children reflected local infection burdens [22, 23]. Some studies have shown that household transmission is the primary infection modality in this age group [24]. Taken together, these data show the importance of managing infectious diseases among school-age children during the COVID-19 pandemic [21] and support the need for protecting school-age children from novel and re-emerging pathogen threats.

Near-source sampling (NSS) involves the collection of wastewaters within the wastewater conveyance system or storage network upstream of a wastewater treatment plant (WWTP). For example, samples have been obtained from pumping stations, clusters of buildings, high-risk industrial settings (e.g. meat processing facilities) or at the individual building level [3, 11, 14]. Other forms of NSS include sampling defined populations within buildings, hubs for onward transmission (e.g., transport systems) or closed, confined, or poorly ventilated spaces (e.g., aircraft / ships). NSS has been applied in prisons [25], care homes [26], airports and aircraft [27], cruise ships [28], hospitals [29], quarantine facilities [30] and academic halls of residence [31], among other congregate locations. Localised and NSS generates data with a spatial focus supporting implementation of targeted public health measures e.g. clinical testing, the use of face masks, surge vaccination, partial closure of schools, case isolation, improved ventilation and targeted pharmaceutical interventions [32].

The current research explores the potential for school wastewater monitoring to provide an independent, actionable data stream to inform public health planning on COVID-19 as well as other infectious disease threats. To test this, wastewater was collected up to four times a week (Monday-Thursday) in 16 schools within England (U.K.), enabling data collation to take place over an academic year. School wastewater testing for fragments of SARS-CoV-2 occurred throughout infection waves driven by several SARS-CoV-2 variants of concern (VoC), including the SARS-CoV-2 wild type, Alpha and Delta variants. A subset of wastewater samples was sent for genomic sequencing, enabling the determination of the presence of SARS-CoV-2 and the nature of the variant(s) contributing to COVID-19 infections within schools. A further sub-set of samples were screened for norovirus by qPCR, and a further >280 microbial pathogens and >1200 antimicrobial resistance (AMR) genes were assayed using respiratory pathogen enrichment followed by deep metagenomic sequencing to consider the utility of these additional targets to further inform on health threats within the schools. Together the utility of combining these data types (viral loading, novel targets from NSS school wastewater) with community wastewater and clinical infection data to help design effective disease surveillance systems in schools, is explored.

## Methods and materials

### Sample sites

Wastewater samples were collected at 16 schools over 31 weeks (excluding term breaks and holidays) between 20 October 2020 and 15 July 2021. In total, 10 primary schools (students aged 4–11 years), five secondary schools (students aged 12–18 years) and one further education school (student age >16 years) were included in the study. Schools were selected across four local authorities, one in the South East (A1), one in the North West (A2) and two in the South West (A3, A4). Selected schools encompassed different levels of deprivation (defined by the quintiles of the Index of Multiple Deprivation, a measure of relative deprivation based on a wide range of an individual’s living conditions) [33] and diversity (based on the percentage of pupils classified as white British as published by school census) (School Census Pupil Level—GOV.UK (education.gov.uk)). The 16 schools selected were located in both high and low prevalence areas, based on confirmed cases of COVID-19 infection at the beginning of the study (S1 Table).

**Fig 1:**
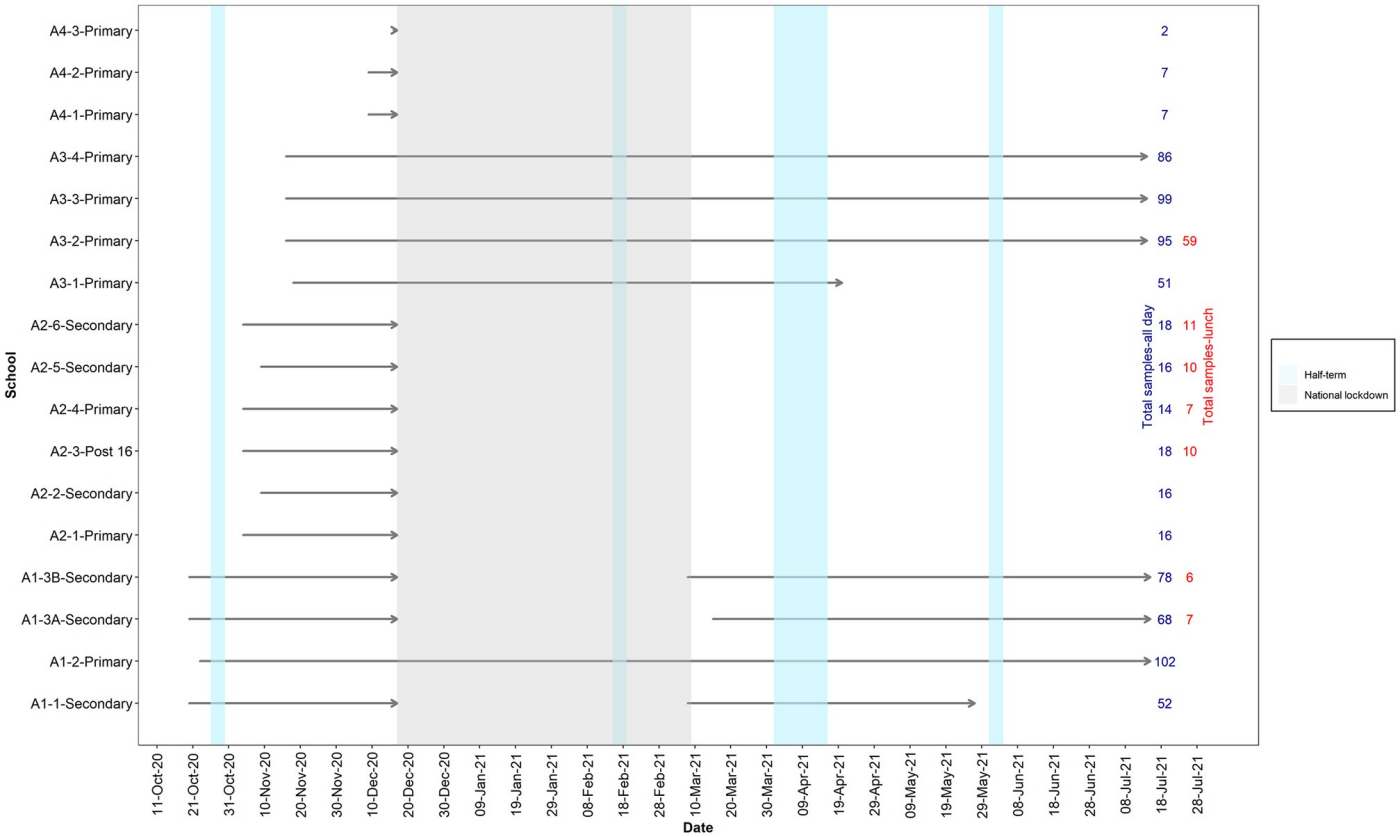
School overview and sampling timeline during 2020/2021 school year. The grey area represents the national lockdown which included school closures across England which excluded key worker children who were exempt. Schools continued to be sampled if student attendance was over 100 pupils. South East: Area 1 (A1); North West: Area 2 (A2); South West: Area 3, Area 4 (A3, A4).

During the monitoring period, the emergence of the Alpha VoC (known as the second wave of the pandemic in the UK) resulted in a subsequent lockdown and national closure of schools (week commencing 04 January 2021 to 07 March 2021, inclusive). During the national lockdown, schools remained open for specific targeted groups i.e., children of key workers. Where student number were <100, sampling was halted to avoid concerns about identifying sampled individuals within this potentially vulnerable population. Therefore, five schools attended by >100 students continued to be sampled throughout the national lockdown, thus continuously sampled throughout the monitoring period. In March 2021, schools reopened, and sampling was reinstated in two of the original 16 schools after consultation with local and national public health decision-makers and school management (Fig 1).

### Sample collection

Comprehensive site surveys were undertaken as part of an earlier trial to identify sample locations [21]. To enable effective sample collection from school sewers, a small weir was created from a brick placed in the wastewater pipe to generate a pool of wastewater to assist sample collection during low flow periods. Samples were collected using an Aquacell P2-COMPACT (Aquamatic) autosampler as 7-hour composites (one-minute sampling in every five minutes; from now on referred to as ‘day sampling’), situated at the first available location downstream of all the drains emerging from the school. An additional autosampler was co-located in several schools (Fig 1) to run at a higher sampling rate of one-minute sampling in every two minutes to target the lunch period (12pm-2pm) (from now on referred to as ‘intensive sampling’). Sampling started on 20 October 2020, initially twice a week, increasing to four times a week from 4 November 2020. The wastewater was collected from each site daily and transferred into plastic 5-litre containers (polypropylene or polyethylene terephthalate) and immediately transported on melting ice to the laboratory for further processing (determination of basic wastewater characteristics and viral concentration/RNA extraction), and an aliquot extracted for cryogenic preservation (-80°C).

### Wastewater analysis

The wastewater was analysed for total suspended solids (TSS), ammonium (NH_4_-N), orthophosphate (PO_4_-P), total chemical oxygen demand (tCOD), soluble chemical oxygen demand (sCOD), pH, conductivity and dissolved oxygen, all following standard methods [34]. Viral concentration and RNA extraction were undertaken according to the polyethylene glycol (PEG) precipitation method [35] without modification [21, 36]. SARS-CoV-2 was detected using quantitative Reverse Transcription—Polymerase Chain Reaction (RT-qPCR), targeting nucleoprotein 1 (N1 gene) and the envelope protein (E gene) [35]. The Limit of Detection (LOD; 1268 Gene Copies (GC) / L for N1 and 2968 GC / L for E) and the Limit of Quantification (LOQ; 9196 GC / L for N1 and 21300 GC / L for 21300 GC / L for E) were reported previously in the pilot study [21]. Samples were accepted as positive detections if above or equal to the relevant LOD values. The % positivity rate was defined as detecting one or both SARS-CoV-2 genes above LOQ over a defined period.

### Detection of human Norovirus

Extracted RNA samples were pooled per week per site and tested for the presence of human Norovirus using primers from the Norovirus Genogroups I and II Advanced kit (Genesig, Primer Design), which targets the capsid protein gene (GI) and RNA dependent RNA polymerase gene (GII). Primer and probe sequences are proprietary and available from the manufacturer on request. Detection was by RT-qPCR performed using the QuantStudio^™^ 7 Pro and analysed by the Design and Analysis software v2.5 system. The PCR reaction mixtures at 20 μL were prepared using Oasing^™^ Lyphophilised OneStep 2x RT-qPCR Master Mix (Primer Design^™^) per the manufacturer’s instructions. The PCR efficiency was accepted at 90% and above.

### Recovery testing using wastewater phage spiking

Wastewater supernatants (i.e. solids and biomass removed; see pilot study [21] for method) were spiked with the *Pseudomonas* virus Phi6 (Φ6; 7.5x106 GC), which acted as a process control to evaluate RNA extraction efficiency. 233 wastewater samples (27.2% of all samples collected) were spiked with Phi6 (DSM-21482), which was propagated using the conventional double-layer agar method and its host *Pseudomonas syringae* (DSM-21518), provided by Dr Kelly Jobling (Newcastle University, UK). Phage titre was estimated by counting plaque-forming units (pfu/mL) and confirmed using RT-qPCR. Extraction of the phage involved overnight culture overlay with sterile PBS (4 mL) at 4°C. The supernatant from the plate was aspirated, filtered through a 0.45 μm sterile filter and stored at 4°C before being used to spike wastewater samples. Aliquots of Phi6 were stored at -80°C and defrosted as needed. The recovery of Phi6 was determined by RT-qPCR (QuantStudio 7 Pro) using the Oasing Lyphophilised OneStep 2x RT-qPCR Master Mix (Primer Design) and the same thermocycler conditions used for Norovirus detection. Phi6 was detected and quantified using specific primers and probe sequences [37] purchased from ThermoFisher. The primer set; forward 5’-TGGCGGCGGTCAAGAGC-3’; reverse 5’-GGATGATTCTCCAGAAGCTGCTG-3’ and the probe 5’-FAM-CGGTCGTCGCAGGTCTGACACTCGC-MGB-NFQ-3’ corresponded to the amplification region (cDNA fragment of 232 bp) within the phi-6S1 gene, located on the S segment and coding for the P8 protein. Phi6 plasmid controls consisted of DNA fragments reconstructed by GeneArt Strings (450 bp, sequence within the phi-6S1 gene). The plasmid was resuspended and log serially diluted (5x10^6^ - 5x10^0^ copies/reaction) for use as the standard curve. The recovery of Phi6 was calculated using the following formula:

$$Recovery\;\%=\left(average\;GC\;per\;reaction\;\times \;DF\;\right)÷\left(Initial\;spike\;of\;extraction\;control\right)\times 100$$

DF = Dilution Factor; 0.05ml of RNA sample aliquoted from each 0.1ml elute = DF of 20

Initial spike of extraction control = 7.5x10^6^ copies of Phi6.

SARS-CoV-2 genome copies were not corrected based on Phi6 recovery in line with convention in wastewater monitoring studies and therefore represent uncorrected values. The current Phi6 recovery was used to assess the consistency of extractions, not as a tool for normalisation.

### SARS-Cov-2 variants of concern

277 samples were selected for amplicon sequencing; the majority were N1 positive (N = 239, 86.3%) with an additional subset of samples (N = 38, 13.7%) which presented below the LOD but had evidence of amplification. Purified RNA was cleaned with Mag-Bind TotalPure NGS beads (Omega Bio-Tek) and reverse-transcribed using the LunaScript RT SuperMix Kit (New England Biolabs) with the following thermocycler conditions: 25°C for 2 minutes, 55°C for 45 minutes, 95°C for 1 minute and hold at 4°C. Library preparation was undertaken using EasySeq^™^ RC-PCR SARS CoV-2 (novel coronavirus) Whole Genome Sequencing kit v3.0 (NimaGen). Amplicons had a further round of cleanup (as above), and sequencing was generated at 2×150bp paired-end reads using an Illumina NovaSeq 6000 (Illumina). The bioinformatics pipeline used has been previously described in detail [38]. In brief, raw sequence reads were processed using the *ncov2019-artic-nf v3* pipeline (https://github.com/connor-lab/ncov2019-artic-nf). Subsequently, VarScan was used to identify Single Nucleotide Polymorphisms (SNPs) and insertions/deletions (Indels), which were filtered against signature mutations of VoCs circulating at the time of the study using a custom script. SARS-CoV-2 VoC profiles were generated based on designations from Public Health England (https://github.com/phe-genomics/variant_definitions) and case definitions, i.e. confirmed or possible presence of a VoC, were designated to wastewater samples based on the number of their SNPs detected as follows:

B.1.1.7 (VOC-20DEC-01) Alpha was considered to have 13 signature SNPs; the variant was reported as confirmed if ≥ 10 of 13 signature SNPs were detected, possible if ≥ 5, and no detection if ≤ 4.
B.1.617.2 (VUI-21APR-02) Delta was considered to have 13 signature SNPs; the variant was reported as confirmed if ≥ 9 of 13 signature SNPs were detected, possible if ≥ 5, no detection if ≤ 4.
Delta substrains AY.43, AY.46.5 and AY.2 were reported as confirmed if ≥ 70% of SNPs in respective profiles were detected, possible if ≥ 50%, no detection if ≤ 50%.

A “Confirmed” status was thus assigned to samples with a high degree of confidence that a variant was present, while a “Possible” status was assigned if there was some evidence of a variant being present. Logistic regression was applied to analyse the relationship between N1 and E GC/mL detection in wastewater (predictor variables) and the probability of identifying SARS-CoV-2 VoC (response variable). Additionally, multinomial logistic regression was performed to assess the relationship between N1 and E gene copies in wastewater samples and whether a variant was confirmed as Alpha or Delta or not detected (based on the above definitions).

### Wastewater monitoring to screen for targets of clinical significance within schools RPP-AMR

To explore wastewater monitoring for additional public health biomarkers, selected samples (n = 59, pooled bi-weekly n = 8) (from A3-3-Primary) were screened for other targets, including pathogens and antimicrobial resistance (AMR) genes. RNA and DNA extracts were pooled over two-week periods in an effort to reduce noise in the data generated by the stochasticity of people attending school and using the toilet. In total, >280 pathogens, including respiratory bacteria, virus, and fungal targets, alongside >1200 AMR alleles, were screened via a Respiratory Pathogen ID/AMR (RPP-AMR) Enrichment Kit (Illumina) and metagenomic sequencing, performed by Molecular Research LP (MR DNA lab, Texas, USA). The process included the conversion of RNA to cDNA by reverse transcription, followed by tagmentation and amplification. PCR was used to amplify the enriched fragments; the DNA library was evaluated using a Qubit and an Agilent 2100 Bioanalyzer (Agilent Technologies). Libraries were pooled in equimolar ratios and sequenced using the NovaSeq 6000 for 76 cycles. Cloud-based software CosmosID was used to assign taxa and sequences from the read data. In brief, unassembled reads were compared to a curated database to analyse unassembled reads, including for pathogens and AMR targets.

### COVID-19 case rates and wastewater viral loads

Two schools (A3-2-Primary and A3-3-Primary) were selected to investigate the relationship between SARS-CoV-2 detection within the school wastewater, SARS-CoV-2 detection at the receiving WWTP and local community COVID-19 case data. The schools were selected as both were sampled throughout England’s national school lockdown with relatively high levels of positivity. Wastewater data from the local treatment plant was acquired from the UK Government’s Wastewater Monitoring programme for England [39, 40]. Age-stratified daily COVID-19 cases at the community level were obtained from the UK Health Security Agency (UKHSA). The latter were used as clinical testing data was unavailable from schools in England during the study. Positive signal (measurements above LOD) and SARS-CoV-2 N1 gene concentration in wastewater were used for modelling (local polynomial regression, LOESS) the NSS detection trends for the school case studies and WWTP with overlap to school catchment area. Community COVID-19 cases were visually compared to the SARS-CoV-2 wastewater signal trends. A lead/lag analysis was performed for the SARS-CoV-2 concentrations (GC/L) in school wastewater and local WWTPs to compare the signal between the two-time series using the Pearson’s correlation coefficient to assess the degree of temporal correspondence between them.

### Ethics

Permission to access sites for wastewater collection was obtained from the Head Teacher of each school. Prior to sampling commencing at each site, a specialist wastewater sampling company (Aquaenviro) undertook a risk assessment method statement (RAMS). Ethical approval for this study was obtained from the Middlesex University Ethics Committee (14795).

## Results

### SARS-COV-2 detection in school wastewater

A total of 855 wastewater samples were analysed for SARS-CoV-2 RNA. Overall, SARS-CoV-2 amplicons (as N1 and/or E) were detected in 31.8% (N = 272) of samples. Of these 272 samples, the N1 target sequence was present in 242 samples (89%) and the E gene in 120 samples (44.1%), with both genes present in 90 samples (33.1%). 152 samples were positive for the N1 gene only, while 30 were positive for the E gene only (S1 Fig). The number of gene copies detected ranged from 1.3x10^3^ GC/L (LOD) to 9.2x10^6^ GC/L for the N1 gene and from 3.0 x10^3^ GC/L (LOD) to 1.3x10^6^ GC/L for the E gene (S2 Table). The lowest RT-qPCR CT values for N1 and E were 26.74 and 27.43, respectively. The mean Phi6 recovery was 7.5% (0.01–80.87%), similar to the recovery rates reported for other studies in the literature (e.g. 2.1%-33.7% [41], 0.078%-0.51% [42] and 1.4%-3.0% [43]). None of the targets measured in the wastewater had a significant positive correlation (p > 0.05) with recovery of the viral surrogate (S3 Table).

The SARS-CoV-2 positivity rate (i.e., percentage of samples in which N1 and/or E genes were detected in relation to the total number of samples collected) was 20% in the first week of sampling (week starting 20 October 2020), with an overall positivity rate of 18.5% for samples collected over the following three-week period (4–25 November 2020). The highest positivity rate was 80.4% (week commencing 30 November 2020), with frequency of detection falling to 56% and 50.6% in the penultimate and final week of the autumn term, respectively. At the end of the autumn term (three weeks before the Christmas vacation), both N1 and E genes were detected across all samples collected from six schools across the three areas. During the period of national school closure, the positivity rate in the five schools where monitoring continued (four primary and one secondary) was 20.6% (11 January—11 February 2021) (S2 Fig).

In England, schools reopened from 8 March 2021 (Fig 1) and the overall sample positivity rate increased from 20.6% (beginning of March 2021) to 29.3% (end of March 2021; start of the Easter vacation). During term time between the Easter break and May half-term break (19 April 2021–27 May 2021), the positivity rate was 28.2%. During the summer term (08 June 2021–15 July 2021), 34.6% of the samples were positive, primarily with a single target (N1 or E gene) detected. In two schools, there were pronounced periods of increased concentrations within wastewater after the May half-term break. Over two days in School A1-2-Primary (08 June 2021 and 09 June 2021) levels of N1 and E genes were above 1.81x10^4^ and 6.4x10^3^ GC/L, respectively. Higher concentrations were reported at A3-2-Primary, where N1 levels up to 9.2x10^6^ GC/L were detected between 10 June 2021 and 06 July 2021 (S2 Fig).

### Establishing link between COVID-19 infections cases and wastewater data

**Fig 2:**
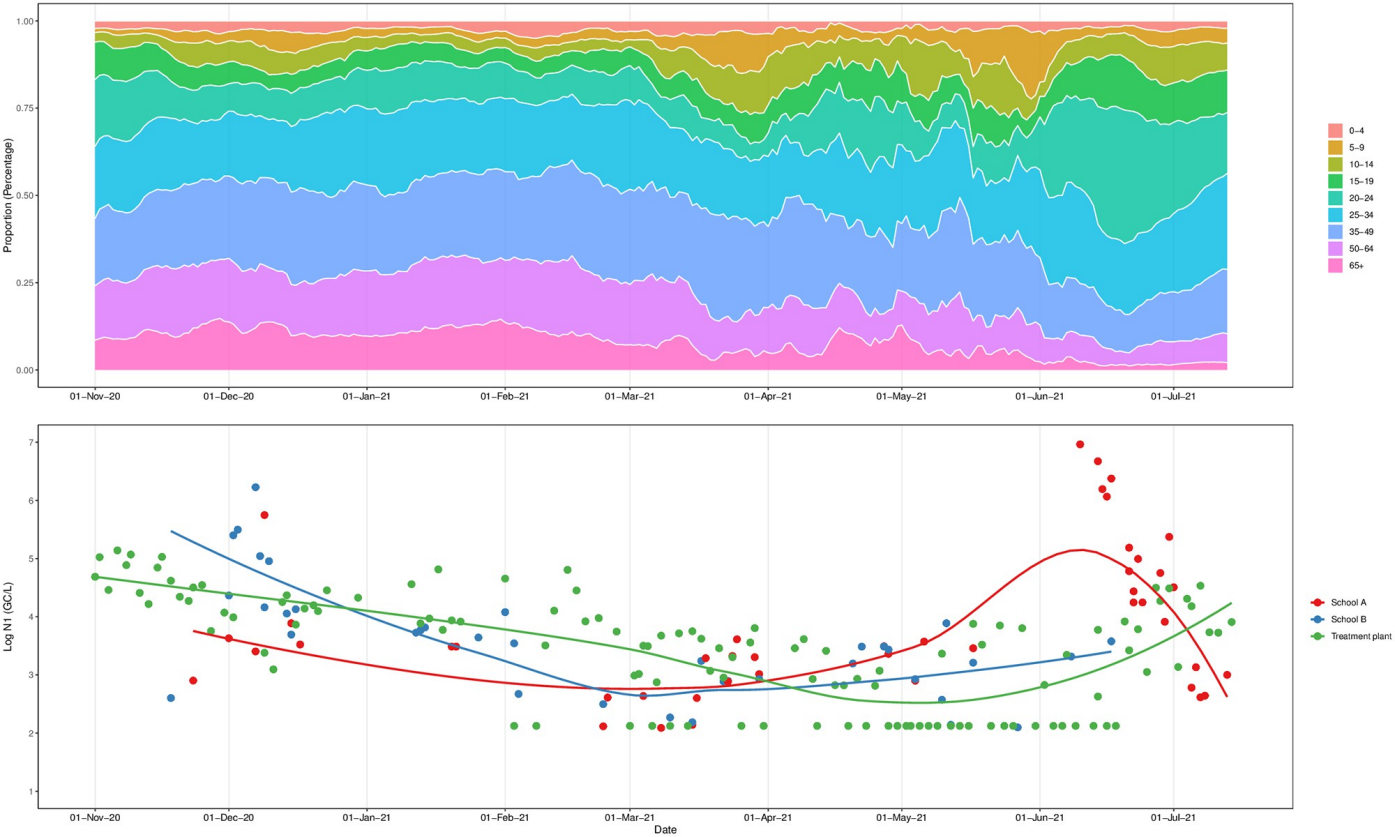
Age-specific clinical COVID-19 cases in A3 (expressed as a % of population) (top). SARS-CoV-2 wastewater from schools and the (associated) WWTP (bottom).

The analysis of selected school wastewater data (A3-2-Primary and A3-3-Primary) with data derived from the receiving WWTP shows levels of SARS-CoV-2 concentration (GC/L) in wastewater in schools and associated WWTP were elevated (comparison to previous months) from November 2020 to January 2021, showing a steady 5% decline during the January—March 2021 lockdown. Following school reopening in March, SARS-CoV-2 concentrations showed a 24.3% and 18.9% increase in both schools, respectively, with the detection of an outbreak at the end of May 2021 (confirmed by the school; personal communication). The lead/lag analysis between SARS-CoV-2 schools and WWTP concentration shows a maximum correlation when school data are lagged by two weeks (Pearson’s correlation coefficient 0.51, p<0.01) for A3-2-Primary and (Pearson’s correlation coefficient 0.40, p = 0.01) A3-3-Primary, suggesting that the signal in school wastewater precedes the increase in the concentrations reported at the WWTP in these cases (S4 Table). The highest SARS-CoV-2 concentration determined in this study was reported for A3-2-Primary during this time period (9.2 x 10^6^ GC/L) compared to concentrations observed in the same time period in the WWTP (Fig 2 - bottom panel).

Clinical case data indicated that from October 2020 to December 2020, COVID-19 cases among school-aged children (5–19 years) in the community (MSOA level) surrounding the two schools contributed just under 20% of the total cases, decreasing to 15% during the January—March 2021 lockdown. As soon as schools reopened on 8 March 2021, school-aged children contributed over 30% of the cases recorded. After a decrease during the second half of April (associated with the Easter vacation period of school closure), cases again increased from the end of May to June 2021, with the largest contributor to total case numbers identified as the primary school age group (5–9 years old). This pattern in the COVID-19 cases in school-aged children aligned with trends in SARS-CoV-2 concentration is the school wastewater (Fig 2 - top panel).

### Wastewater characteristics variability between and within schools

No statistically significant relationship was determined between any measured wastewater parameter and SARS-CoV-2 concentrations at any school. One exception was for ammonia, which showed a weak correlation with the SARS-CoV-2 N1 gene (R2 = 0.28; p = 0.01) at A3-2-Primary under a high prevalence scenario (i.e., a period when SARS-CoV-2 was detected daily at concentrations >LOQ). Under low prevalence scenarios (i.e., infrequent SARS-CoV-2 detection), this relationship was no longer significant (p>0.05). A similar pattern was also observed in the second case study school (A3-3-Primary) but the relationship was not significant. In contrast the relationship between pH and N1 gene concentrations varied in direction and strength between case study schools (S3 Table). Overall, there was no correlation between student population and PO_4_-P/ NH_4_-H concentration. Kitchen space and its use for lunch preparation was recorded for each school to assess the contribution of kitchen waste as an additional source of ammonia (S5 Table). A welch t-test was applied to account for unequal sample size between schools which had lunch preparation onsite and those without, which showed no significant difference for NH_4_-H (t = - 1.2944, p = 0.21) and PO_4_-P (t = - 0.9881, p = 0.33) (S5 Table) therefore kitchens did not contribute to the loading of key wastewater constituents often used for assessing population equivalent. Therefore, other factors such as irregular toilet behaviour or possibly chemical cleaning reagents could have contributed to lack of relationship between SARS-CoV-2 and these wastewater parameters.

### Wastewater SARS-CoV-2 variant sequencing

**Fig 3:**
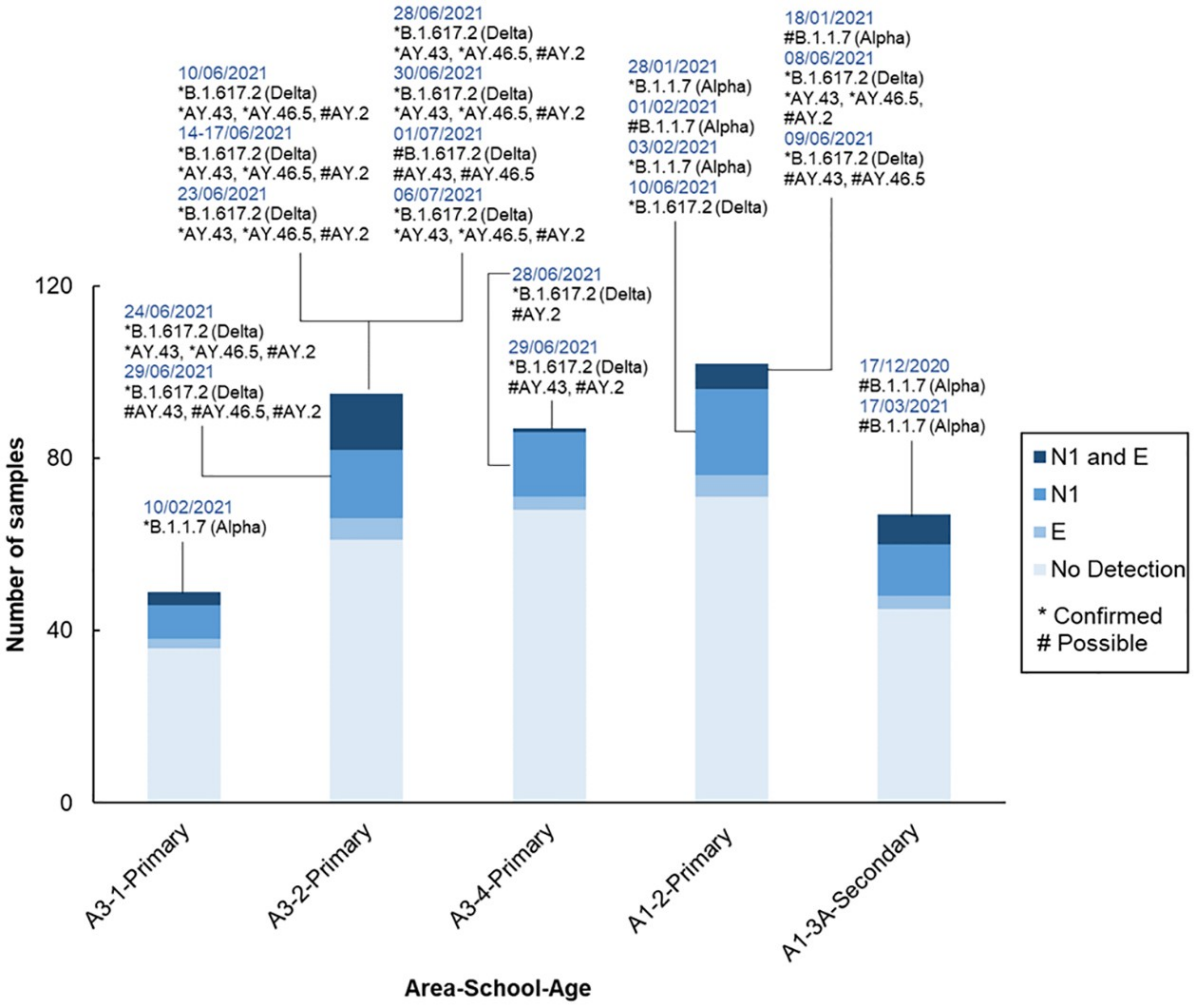
SARS-CoV-2 variants from school wastewater. The presence of the variants of concern and variants under investigation (as classified during the monitoring period) in the wastewater samples were identified based on the number of their signature SNP mutations. (*) Confirmed—All or majority (more than ∼70%) of signature SNPs detected. (#) Possible—Detection of more than 38% (Alpha and Delta) or 50% (Delta substrains) of signature SNPs.

The detection of VoC and variants under investigation (VuI) was reported as confirmed or possible based on the number of signature SNP mutations. Alpha (B.1.1.7) was detected as possible on 17 December 2020 at A1-3A-Secondary, with confirmed cases in A1-2-Primary school on 28 January 2021 and 03 February 2021 and in A3-1-Primary school on 10 February 2021. The final detection of Alpha, a possible detection, was at A1-3A-Secondary on 17 March 2020. The first confirmed case of Delta (B.1.617.2) amongst the monitored schools was at A1-2-Primary school on 08 June 2021, a signal that persisted for three consecutive days (08–10 June 2021). A spike in the presence of Delta was observed in A3-2-Primary school for almost a month, from 10 June—07 July 2021. Delta’s sub-lineages AY.43, AY.46.5, and AY.2 were also detected in Delta-positive school wastewater samples (Fig 3). Indeed, when Delta and its sub-lineages were found, detections were predominantly confirmed.

Logistic regression showed that an increase in N1 and E gene detection in wastewater increased the likelihood of VoC detection significantly [N1: Odds Ratio (OR) = 1.00776, p = 0.01; OR = 1.0003226, p≤0.001, S6 Table). Similarly, when multinomial logistic regression was applied, an increase in N1 and E gene detection increased the likelihood of both Alpha variant (N1: OR = 1.00708, p = 0.03; E: OR = 1.00029; p = 0.007) and Delta variant detection (N1: OR = 1.00825; p = 0.009; E: OR = 1.00034; p = 0.002, S7 Table).

### Screening for other disease markers in school wastewater

Weekly pooled wastewater samples were tested for Norovirus, with infrequent and low-level detection reported using RT-qPCR from March to July 2021 (Table 1). The target (RNA-dependent RNA polymerase gene), which screens specifically for the GII genogroup of Norovirus, was quantified at 2380 GC/L (Ct = 33) in a pooled sample (29 March 2021–31 March 2021) from A1-2-Primary, with negligible detection of Norovirus GI (Ct = 42; 12.41 GC/L) in A1-3B-Secondary (pooled sample 28 June 2021–01 July 2021).

**Table 1:** Detection of norovirus target in weekly pooled school wastewater samples by RT-qPCR.

School	Pooled samples (date)	Norovirus gene targets	CT	Gene concentration (GC/L)
A1-2-Primary	29–31 March 2021	GII	33.02	2380.33
A1-3B-Secondary	28 June-1 July 2021	GI	42.36	12.41

GI-capsid protein gene; GII-RNA dependent RNA polymerase gene

To further explore the value of wastewater monitoring in schools, a range of clinically relevant targets were examined using a novel RPP-AMR enrichment approach. The RPP-AMR panel enriches for >280 respiratory pathogens, including SARS-CoV-2, influenza, a range of viruses, bacteria, fungi and more than 2000 AMR marker genes. Although DNA/RNA quantities used for the assay were consistent with the manufacturer’s specifications, the results were uniformly consistent in only identifying the presence of a very small number of targets, including: SARS-CoV-2, human Adenovirus 1 and *Cronobacter sakazakii*. SARS-CoV-2 was intermittently detected in samples also testing positive by qPCR, while human Adenovirus 1 (HAV-1) was found within sample A3-3-Primary school sample (4 May 2021–13 May 2021). HAV-1, is a pathogen that is implicated in respiratory infections in young children. The pathogenic bacterium *Cronobacter sakazakii* was detected consistently in pooled A3-3-Primary samples obtained from 22 March 2021–27 May 2021 (Table 2). This pathogen is most frequently associated with contaminated infant formula, causing bacteraemia, meningitis and necrotizing enterocolitis. Although *C*. *sakazakii* was not present on the panel (RPP-AMR), it was still detectable using metagenomic analysis of the raw data generated from the sequencing.

**Table 2:** Detection of respiratory pathogens in pooled wastewater school samples by enrichment and sequencing.

Pooled samples	Sampling period	Pathogens	
		Virus	Bacteria
1	18/01/2021–28/01/2021	SARS-CoV-2	
2	01/02/2021–09/02/2021	SARS-CoV-2	
3	22/02/2021–04/03/2021		
4	08/03/2021–18/03/2021		
5	22/03/2021–31/03/2021		*Cronobacter sakazakii*
6	20/04/2021–29/04/2021		*Cronobacter sakazakii*
7	04/05/2021–13/05/2021	Human Adenovirus 1	*Cronobacter sakazakii*
8	17/05/2021–27/05/2021		*Cronobacter sakazakii*

Pooled samples bi-weeks of A3-3-Primary school

**Fig 4:**
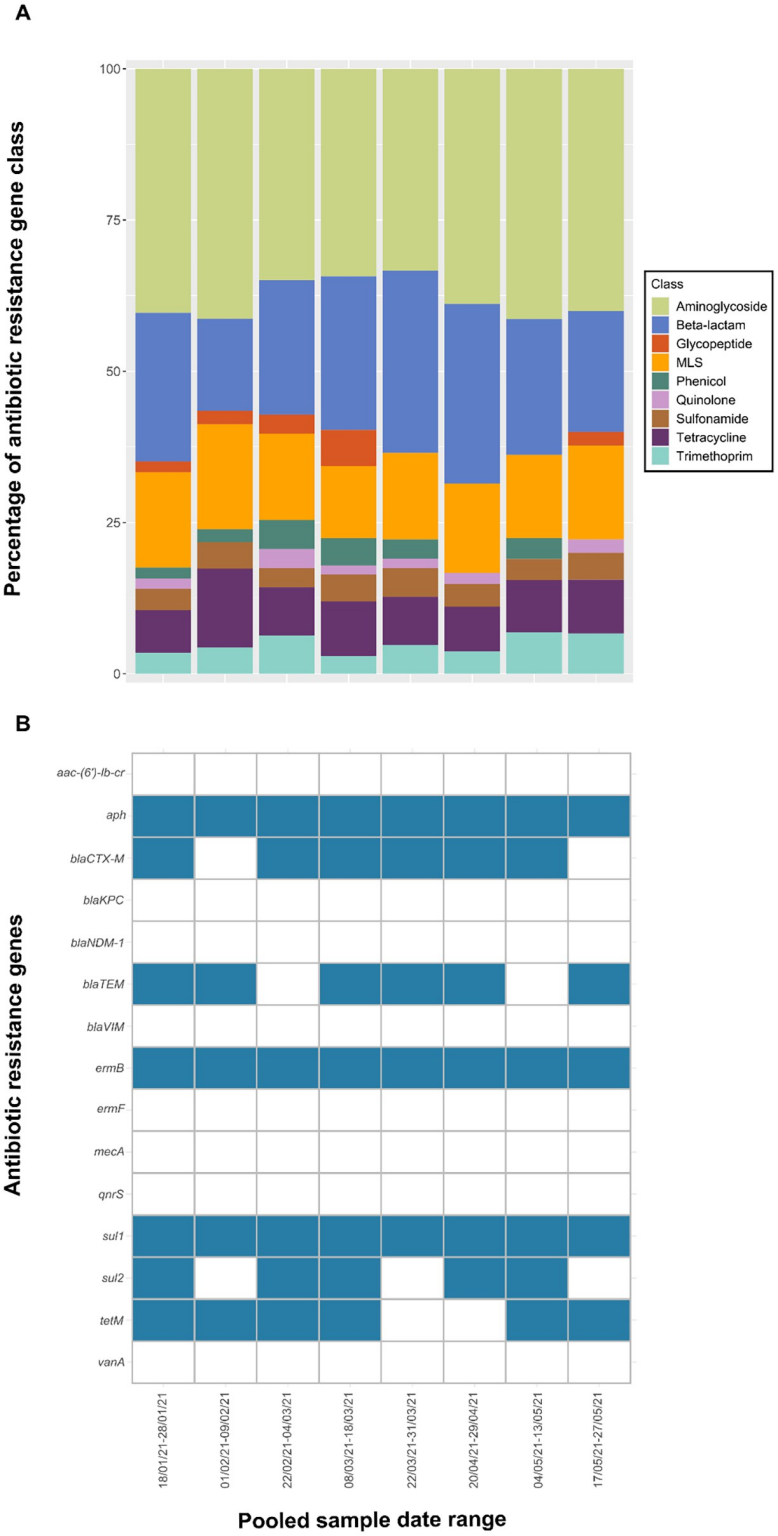
A) Diversity of antibiotic resistance genes by antibiotic class detected within school wastewater. B) Overview of the presence/absence of 15 antibiotic resistance genes identified as key for environmental monitoring within pooled school wastewater samples.

The RPP-AMR also reports on >200 antibiotic resistance genes (ARGs). The abundance of ARGs was clearly higher than those of the pathogens, which might be expected—as such, the presence/absence of these genes has been reported in Fig 4. The relative abundance data was compared between pooled weekly samples to derive the diversity of unique ARGs. Examination of sequence data from the RPP-AMR revealed that genes that were enriched were detected alongside high abundance genes, which were not subject to enrichment. Unique ARGs were displayed by antibiotic class and the resistome diversity was compared over a 16-week period using 2 weeks sample composites within 1 school 8 pooled (A3-3-Primary; Fig 4A).

The presence/absence of 15 ARGs (identified as valuable for environmental resistome monitoring due to their frequency of detection, link to human disease and potential for horizontal gene transfer between environmental and clinical bacteria) [44] in pooled school wastewater samples is shown in Fig 4B. Three of these ARGs were detected in all school wastewater samples: *aph* (conferring aminoglycoside resistance), *ermB* (conferring macrolide resistance) and *sul1* (conferring sulphonamide resistance). In contrast, eight of the target ARGs were not detected in any samples: *aac-(6’)-lb-cr* (aminoglycoside resistance gene); *mecA*, *blaKPC*, *blaNDM-1* and *blaVIM* (beta-lactam resistance genes); *ermF* (macrolide resistance gene); *qnrS* (quinolone resistance gene); and *vanA* (vancomycin resistance gene). The remaining shortlisted genes were detected intermittently throughout the sampling period (Fig 4), reflecting relatively low antibiotic use in school age children.

## Discussion

The COVID-19 pandemic has shown the need for increased preparedness of our health systems, highlighting the need to refine alternative tools for monitoring the spread of diseases and the health of the population in general. Wastewater surveillance has been implemented globally [28, 32, 36, 45–47] to support the response to the pandemic, providing a rapid, low-cost, non-invasive method for capturing symptomatic, pre-symptomatic, and asymptomatic cases potentially guiding targeted responses and reducing associated costs (e.g., clinical testing).

This study is a long-term monitoring continuation of a pilot study that established the potential application of wastewater surveillance as NSS to detect SARS-CoV-2 and other health markers from schools. We reported our initial findings of wastewater detection incidents and rates from October 2020 to December 2020 (the mid-stage of the COVID-19 pandemic including the emergence of the SARS-CoV-2 Alpha (B.1.1.7) variant in the UK) [21]. Detection of the SARS-CoV-2 virus provided evidence of the presence of the virus in schools at different stages of the pandemic, that the signal in schools preceded the signal in the reference WWTP and was associated with clinical cases in the community among school-age children.

During the period of national school closure, the positivity rate in the five schools (four primary and one secondary) was <21% suggesting that the December 2021 lockdown—a period of reduced mixing as a non-pharmacological intervention—resulted in fewer COVID-19 infections within school age pupils. The reduction in SARS-CoV-2 signal observed during the last weeks of the autumn term (20^th^ October 2020- 25^th^ November 2020) was likely due to a mix of control measures: isolation following positive testing and implementation of remote learning following closure in some schools. The extent to which infection within schools drives local outbreaks—as opposed to schools reflecting community infection levels–requires additional research, and wastewater sampling could help answer this question more definitively in future emerging or re-emerging pathogen outbreaks, particularly if concurrent testing of household wastewater samples was undertaken. However, the interpretation of the lower positivity rate reported in wastewater is supported by reference to the age-specific clinical case data (Fig 2 - top panel), which indicates a reduction in the percentage of school-age children testing positive at the start of 2021. This lower level of positive infections did not then change further for the duration of this study, suggesting infections within this age group were not increasing compared to overall infections within the community where infection trends were more variable.

Reported results show how NSS has the capability to identify prevalent variants using whole genome sequencing. In addition, higher SARS-CoV-2 GC/L is associated with higher genome coverage. The chance of recovering good quality genomes, and the required diagnostic SNPs and Indels which are distributed across them, was associated with the presence of both N1 and E SARS-CoV-2 genes at high concentrations, which is indicative of a less degraded sample. Therefore, screening wastewater samples via qPCR for multiple amplicon targets, distributed across the SARS-CoV-2 genome, could be a useful approach to select samples where variant identification is more likely or prioritising resources for samples which will provide the most valuable data to public health decision makers. SARS-CoV-2 variant data available from GOV.UK [48] allowed the comparison of local occurrences of variants at WWTP and school levels. The Alpha variant was not reported in data from the WWTP (corresponding school areas), as it was the dominant variant at the time, but this study recovered and identified the Alpha variant in school wastewater between December 2020 and March 2021. The Delta variant was identified at the school level 3–4 weeks after its first detection at the WWTP, possibly indicating a lag between background community transmission and school outbreak. The first Delta detection was on 5 May 2021 in the WWTP of A1, while the first Delta case was identified on 8 June 2021 at the school (A1-2-Primary) level. The WWTP of A3 first detected the Delta variant on 23 May 2021, while the first detection in the school (A3-2-Primary) was on 10 June 2021. This could be a product of the greater granularity NSS provides and the prospect of school’s acting as hotspots with faster responses to local outbreaks to guide public health interventions. This highlights the important benefits of combining wastewater surveillance using WWTP and NSS to identify outbreaks at the different scales and from different populations inherent within a sewage network.

The utility of sampling school wastewater was further examined through analysis of other relevant pathogenic viruses. Here we examined two of the most common genotypes of Norovirus, due to its economic and public health implications and the propensity for outbreaks in schools [49]. In a similar application of the wastewater surveillance technology, Erster et al. [50] was able to isolate and monitor an emerging outbreak of the novel Enterovirus D69 outbreak in Israel using paired NSS and WWTP monitoring. In the current study, our results corroborate the theory that other pathogens were suppressed during the pandemic, driven by lockdowns [51], school closures [52] and improved hygiene practices [53]. Despite the absence of Norovirus, highly prevalent ARGs were detected in samples, which highlights the potential for wastewater surveillance in mapping antimicrobial resistance, although future research is needed to confirm linkages between detection of gene targets and antimicrobial resistance phenotype. Eze et al. [54] isolated a pan-drug resistant *Acinetobacter baumannii* strain from hospital wastewater in South Africa, responsible for both nosocomial infections and opportunistic infections and fatalities. The authors highlight the value of NSS from hospital wastewater for acquisition of this type of sample but also caution as to the risks posed by pathogens within wastewater for the dissemination of disease-causing agents. Nonetheless clinically important information was derived from wastewater and emerging tools such as metagenomics, target enrichment, lab-on-chip style approaches could provide a flexible platform for NSS monitoring to study infections within congregate communities [55].

Diamond et al. [56] suggested wastewater surveillance could offer a sentinel system if expanded to monitor other pathogens and boost public health preparedness. In the UK and USA, public funded schools usually operate with specific geographic catchments for intake. Thus, NSS of school wastewater has great potential to offer the sentinel system suggested by Diamond et al. [56], as the school represents an age stratified snapshot of the local community. This information is useful as a warning for infection risk in congregate settings where a high degree of social interactions occurs [57, 58] and can support specific community public health response by prioritising socially applicable interventions relevant to each local community (i.e., testing and vaccine acceptability) [45]. School-based wastewater epidemiological surveillance provides more spatial granularity than WWTP level. During the period of the current study authors were able to provide specific, timely, and tailored risk mitigation advice to school communities (parents, students, and staff), via liaison with the school leaders. Future work could identify a tiered framework for hierarchy of monitoring for infectious disease agents in schools as suggested by Berry et al. [59] for healthcare, with wastewater acting as a cost-effective early warning to identify hotpots prior to conducting repeated population-based random surveys in year groups in schools. Petros et al. [58] showed the value of collecting social data including Wi-Fi co-location information from university students alongside NSS using wastewater. The authors showed that length and frequency of social interactions alongside participating in high-contact sports were good predictors of COVID-19 infection risk. This study has implications for school age children who are likely to be exposed to similar activities of heighted transmission risk (i.e., sport), potential poor hygiene practices and greater numbers of social interactions than the majority of the adult population.

Consistent with our initial results, this study did not find a strong correlation between the wastewater parameters and SARS-CoV-2 recovery during long-term monitoring. Sweetapple et al. (2021) [46] reported that measurements of ammonium (NH_4_-N) and orthophosphate (PO_4_-P) concentrations have the potential to be used for population normalisation in wastewater monitoring and that both strongly correlated to SARS-CoV-2 recovery. In this school study, there was no strong positive correlation between SARS-CoV-2 detection and either NH_4_-N or PO_4_-P between and within schools. This could be due to the fluctuating number of pupils at school on individual sampling days and unpredictable bathroom behaviour. In addition, SARS-CoV-2 and water parameters are independent variables, and their correlation has been reported as a function of population size [32]. Other external sources—such as kitchen waste—could have also contributed to ammonia and phosphorus loads, and to consider this schools participating in this study were asked to share information on kitchen use during the sampling period. However, correlation between e.g. population and ammonia levels, as well as difference between schools utilising on-site as opposed to off-site kitchens did not show any significance (S5 Table). This suggests that other factors i.e., detergents, metals etc could impact recovery [60]. Other NSS did not use wastewater constituents to normalise / account for population. For example, Acosta et al. [61] normalise N1, N2 and E to Pepper mild mottle virus (PMMoV) gene copies which accounts for human specific biomarkers. Although variability in diet and lifestyle can influence the concentrations reported [62], other studies [4] have used PMMoV suggesting an efficient and acceptable recovery of RNA during RNA extraction as order / ranges are within published values for municipal (i.e., human derived) wastewater. The surfactant concentrations within wastewater can reduce the recovery of SARS-CoV-2 [63]. This is thought to be due to surfactant with virus interaction reducing stability and therefore recovery. Enhanced recovery of SARS-CoV-2 from wastewater-settled solids has also been reported [64–66], although this aspect was not considered as par with this study.

Previously, we demonstrated that viral RNA signal recovery from school wastewater improved when samples were collected throughout the school day (7 hours). This initial finding is confirmed here, with an all-day sampling approach associated with a positivity rate of 35.6% in in comparison to a lunchtime only sampling positivity rate of 11.9% (S1 Fig). This is likely linked to the autosampler missing flush events in NSS when sampling was conducted over a more limited period. Further tracing toilet use through monitoring of flush events or social surveys would help elucidate how toilet habits influence NSS wastewater surveillance data.

Detection of a single viral RNA target is most common during low prevalence scenarios or in wastewater containing degraded RNA which occurs during elevated temperatures [67], different wastewater matrices or during extended storage times [68, 69]. In schools, low prevalence was the most likely reason for low detection/ positivity rates observed as sampling was undertaken close to source and therefore reduced the time for degradation to occur [70]. The wastewater signal in schools is better associated with clinical cases observed in school-ages population than the signal observed in the WWTP, providing better granular information on the circulation of the virus among that specific age group.

The presence of ARGs is a natural phenomenon, but samples enriched for these gene targets have been identified in wastewater from hospitals [71], pharmaceutical industry [72] and multiple environmental matrices [73]. Sets of bi-weekly pooled samples from A3-3-Primary school were selected (based on high positivity and number of samples collected) to be analysed for other health markers in wastewater during the pandemic. It should be noted that the sequencing output obtained in this study is only qualitative and does not provide measurable expression of the genes. Regardless, results were obtained despite concerns with the limitations of wastewater analysis, further showing capability of health markers being detected when using a single wastewater sample.

This suggests that RT-qPCR was a more sensitive approach under conditions tested but a trade-off exists in the type of datasets which can be generated with wastewater between the depth (of detection) and coverage generated by a single sample. For example, other respiratory viruses, such as HAVdetected in the pooled samples, are shed within the faeces and thus compliant for detection using wastewater. Unlike HAV, *Cronobacter sakazakii* (detected in schools) can be isolated from the environment (i.e. sewer derived), as well as from infections within the shedding population (children and staff at school). The current study is not the first study to apply metagenomics to characterise other pathogen / AMR targets from NSS locations. Spurbeck et al. (2021) [29] applied SARS-CoV-2 amplicon and RNA metagenomic sequencing to wastewater isolated from nursing homes. The authors detected microbes known to cause nosocomial infections, gastroenteritis, cystitis, pneumonia, septicemia, and tetanus. Opportunistic pathogens including *A*. *baumannii*, *A*. *junii* SH205, and *A*. *Iwoffii* SH145 which was similar to Eze et al. [54] and *Campylobacter jejuni*. *jejuni*, which is strongly implicated in gastroenteritis etiology alongside strains of *Escherichia coli* known to cause urinary tract infection. This demonstrates additional value, which can be obtained using wastewater monitoring approaches for infectious disease monitoring and outside of pandemic as numerous clinically relevant disease-causing agents have now been tracked. It is suggested that vulnerable populations (schools, prisons, care homes) and low income settings are particularly amenable to this inexpensive, aggregate, anonymous, scalable and non-invasive approach [3, 74, 75].

The use of NSS within schools raised several challenges, including the understanding and use of data on bathroom usage habits of school-age children (e.g., reliable access to toilets and use levels at schools) and the limitations of the current generation of sample collection technologies [76]. These limitations include access to sampling points within the school environment (both in terms of full characterisation of contributing sources and the opportunities to locate samplers on school grounds) and the mistiming of sample collection in relation to flush events, that could result in limited capture of the viral load transported within the wastewater. In addition, the contribution of each SARS-CoV-2 RNA shedding route (e.g., urine, sputum, faeces) to RNA loads in wastewater has yet to be elucidated in a school (or any other) environment [77]. Despite these limitations, the ability to detect viral SARS-CoV-2 RNA in the highly variable wastewater quality streams expected at a school building level was first demonstrated by Castro-Gutierrez et al. [21]. Finally, tracking mobile workers (i.e., support / substitute teachers / cleaners etc) through passive wastewater monitoring poses inherent challenges and apportioning infections attributed to staff and students and identifying social determinants of risk represents a logical next step for future research. Some of the identified benefits of schools-based wastewater monitoring include generation of longitudinal data sets on the emergence and spread of variants and providing direct information on the transmission in high-risk environments, such as classrooms. Combined with other socio-economic data, wastewater monitoring could indicate schools settings where students, parents, and staff are more likely to face structural barriers to vaccination and/or diagnostic testing uptake [45], supporting the detection of emerging variants [78] and provision of information on the transmission in high-risk environments, such as the classroom [79].

## Conclusion

In this study, new data associated with the dynamics of SARS-CoV-2 in school wastewater monitored for one academic year was generated and linked for the first time to both local clinical data and community wastewater. The wastewater data was highly dynamic and lagged the community wastewater and infectious rate results. SARS-CoV-2 positivity rate was detected during national lockdown with restricted school attendance, suggesting transmission still occurred among those populations. In addition, wastewater showed potential in identifying variants prevalent at different phases of the pandemic, providing proof of concept for detecting emerging VoC in schools. Furthermore, wastewater sample enrichment coupled with metagenomic sequencing and rapid informatics enabled the detection of other clinically relevant viral and bacterial pathogens and AMR, revealing the capability of wastewater monitoring to enable public health authorities to develop targeted prevention and education programmes for hygiene measures within undertested communities across a broad range of use cases. This ‘measure once—test many’ approach increases the insights gathered per sample and the cost effectiveness of insights derived. This could lead to interventions on emerging outbreaks and liaison with local public health decision-makers, who could enforce strategies to slow the spread of infection rates in schools and other settings within vulnerable populations.

## Supporting information

S1 Fig Number of positive SARS-CoV-2 gene detections represented in Venn diagram, showing overlap of N1 and E gene targets presence in the samples. 855 of wastewater samples from schools were collected and analysed for SARS-CoV-2 amplicons (N1 and E genes). Overall 31.8% of samples (N = 272) were positive for either one or both SARS-CoV-2 targets.
(TIF)

S2 Fig Heatmap of SARS-CoV-2 gene detection between October 2020 to July 2021 in wastewater from schools in England. Samples were accepted as positive if equal or above Limit of Detection of respective gene targets. The Limit of Detection for N1 = 1268 Gene Copies (GC) / L and E = 2968 GC / L gene targets.
(TIF)

S1 Table School characteristics during different stages of the monitoring period. (DOCX)

S2 Table Overview of qPCR outcome of the target detections in school wastewater. (DOCX)

S3 Table Correlation between wastewater constituents and the viral recovery in the case study schools. (DOCX)

S4 Table Correlation between SARS-CoV-2 concentrations in two schools and wastewater treatment plant (WWTP). (DOCX)

S5 Table Kitchen spaces and kitchen usage in schools as potential contributors of ammonia and phosphate levels in wastewater. (DOCX)

S6 Table Logistic regression of SARS-CoV-2 amplicon level in wastewater and Variant identification. (DOCX)

S7 Table Multinomial logistic regression, relationship between variant detection and gene copy of N1 and E concentrations. (DOCX)

S1 Data (PDF)
