# Supplementary figures and images for: Wastewater monitoring for detection of public health markers during the COVID-19 pandemic: Near-source monitoring of schools in England over an academic year
Source: PLoS One. 2023 May 30;18(5):e0286259. doi: 10.1371/journal.pone.0286259 (PMC10228768; doi:10.1371/journal.pone.0286259)

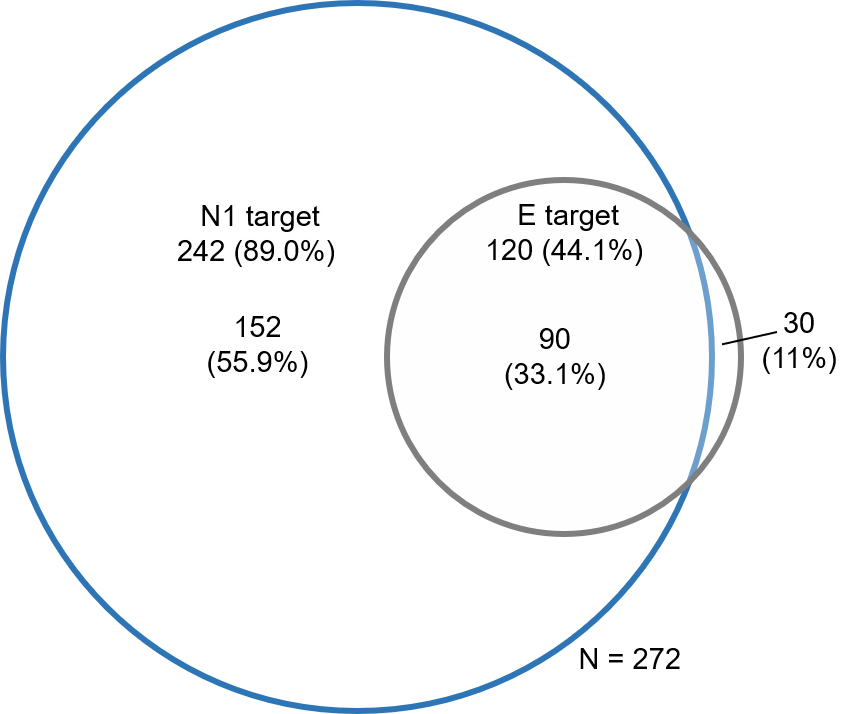

Supplement: S1 Fig — 855 of wastewater samples from schools were collected and analysed for SARS-CoV-2 amplicons (N1 and E genes). Overall 31.8% of samples (N = 272) were positive for either one or both SARS-CoV-2 targets. (TIF) [file pone.0286259.s001.tif]

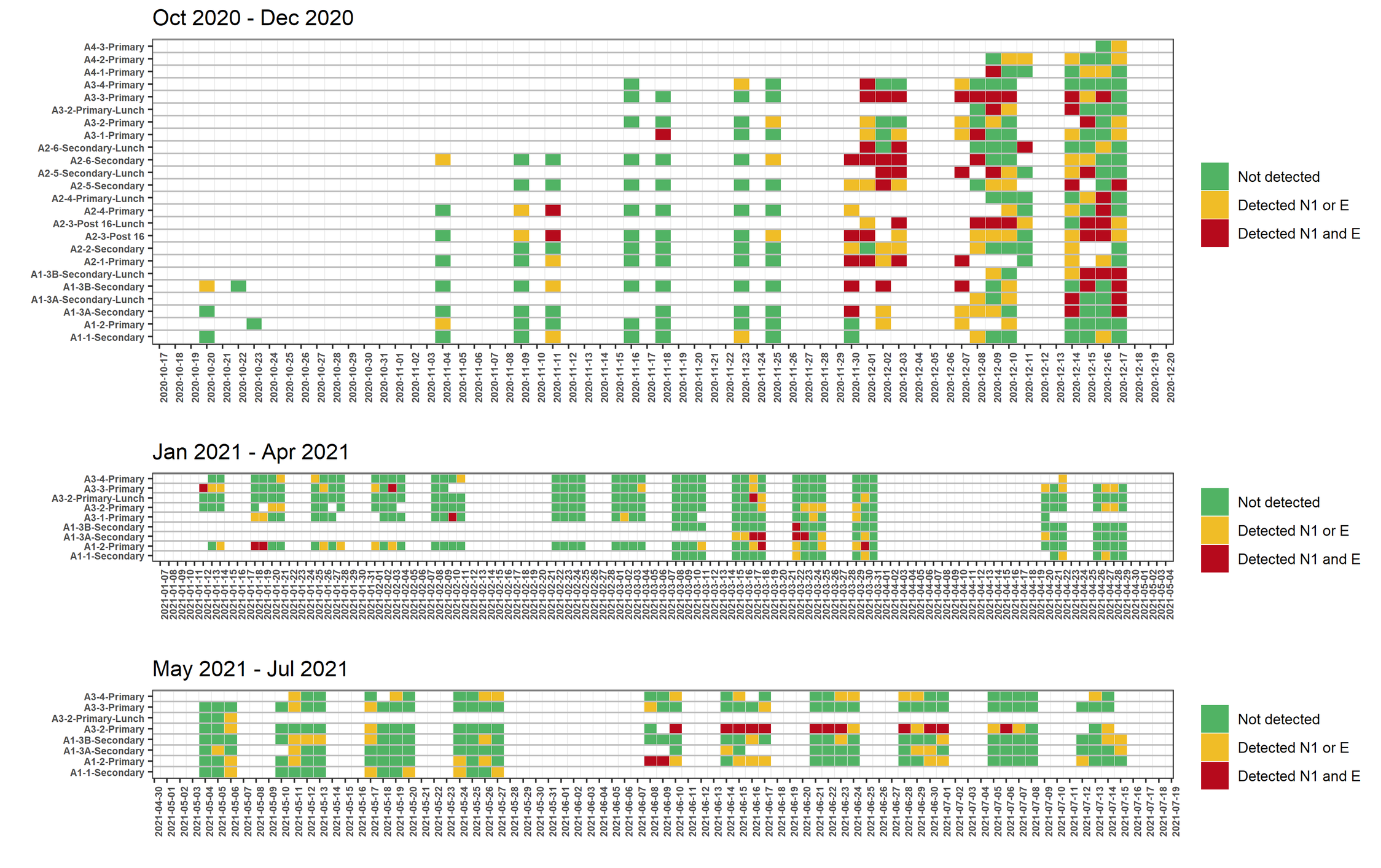

Supplement: S2 Fig — Samples were accepted as positive if equal or above Limit of Detection of respective gene targets. The Limit of Detection for N1 = 1268 Gene Copies (GC) / L and E = 2968 GC / L gene targets. (TIF) [file pone.0286259.s002.tif]
