# Supplementary material for: Wastewater monitoring for detection of public health markers during the COVID-19 pandemic: Near-source monitoring of schools in England over an academic year
Source: PLoS One. 2023 May 30;18(5):e0286259. doi: 10.1371/journal.pone.0286259 (PMC10228768; doi:10.1371/journal.pone.0286259)
Supplement: S1 Table — (DOCX) [file pone.0286259.s003.docx]

**S1 Table. School characteristics during different stages of the monitoring period**

| **Phase of education** | **Pilot duration-20^th^ Oct-17^th^ Dec2020**  **(N of Schools/Students)** | **Lockdown-12^th^ Jan- 4^th^ Mar2021**  **(N of Schools/Students)** | **Schools reopened-8^th^ Mar-15^th^ Jul2021**  **(N of Schools/Students)** |
| --- | --- | --- | --- |
| Primary | 10/3335 | 5/>100 per school* | 5/2045 |
| Secondary | 5/5150 | 0 | 2/2969 |
| Post 16 | 1/ n.a. | 0 | 0 |
| **LSOAs quintiles (IMD)** |  |  |  |
| Q1 - lower | 7 | 2 | 3 |
| Q2 | 3 | 1 | 1 |
| Q3 | 3 | 1 | 2 |
| Q4 | 2 | 1 | 1 |
| Q5 – higher | 1 | 0 | 0 |
| **Pupils classified as white British** |  |  |  |
| Very Low: Up to 20% | 5 | 1 | 3 |
| Low: Between 21% and 40% | 1 | 1 | 1 |
| Med: Between 41% and 60% | 2 | 0 | 0 |
| High: Between 61% and 80% | 4 | 2 | 2 |
| Very High: Between 81% and 100% | 3 | 1 | 1 |
| NA | 1 |  |  |
|  | **Min-Max** |  |  |
| **Number of pupils (official)** | 143-2061 | >100 per school | 187-2061 |
| **COVID-19 new cases rate per 100,000 population (weekly)** | 22.3-3008.2 | 15.1-1579.6 | 15.1-1593.2 |

*children of key workers
