# Supplementary material for: Wastewater monitoring for detection of public health markers during the COVID-19 pandemic: Near-source monitoring of schools in England over an academic year
Source: PLoS One. 2023 May 30;18(5):e0286259. doi: 10.1371/journal.pone.0286259 (PMC10228768; doi:10.1371/journal.pone.0286259)
Supplement: S2 Table — (DOCX) [file pone.0286259.s004.docx]

**S2 Table. Overview of qPCR outcome of the target detections in school wastewater**

| SARS-CoV-2 targets | Minimum CT | Concentration (GC/L) | |
| --- | --- | --- | --- |
|  |  | Max | Min (above LOD) |
| N1 | 26.74  (10/06/2021-A3-2-Primary) | 9.2x10^6^  (10/06/2021-A3-2-Primary) | 1.3x10^3^  (29/03/2021-A1-2-Primary) |
| E | 27.43  (10/06/2021-A3-2-Primary) | 1.3x10^6^  (07/12/2020-A3-3-Primary) | 3.0x10^3^  (11/03/2021-A1-2-Primary) |

Sampling date and site denoted in the brackets
