# Supplementary material for: Wastewater monitoring for detection of public health markers during the COVID-19 pandemic: Near-source monitoring of schools in England over an academic year
Source: PLoS One. 2023 May 30;18(5):e0286259. doi: 10.1371/journal.pone.0286259 (PMC10228768; doi:10.1371/journal.pone.0286259)
Supplement: S3 Table — (DOCX) [file pone.0286259.s005.docx]

**S3 Table. Correlation between wastewater constituents and the viral recovery in the case study schools**

| N1 (GC/L) correlation: | A3-2-Primary | A3-3-Primary |
| --- | --- | --- |
| pH | r = 0.26  p = 0.01* | r = -0.18  p > 0.05 |
| NH_4_-N (mg/L) | r = 0.28  p = 0.01* | r = 0.12  p > 0.05 |
| TSS (mg/L), PO_4_-P (mg/L), Conductivity (μS), tCOD (mg/L), sCOD (mg/L), Dissolved Oxygen (mg/L) | p>0.05 | |
