# Supplementary material for: Wastewater monitoring for detection of public health markers during the COVID-19 pandemic: Near-source monitoring of schools in England over an academic year
Source: PLoS One. 2023 May 30;18(5):e0286259. doi: 10.1371/journal.pone.0286259 (PMC10228768; doi:10.1371/journal.pone.0286259)
Supplement: S4 Table — (DOCX) [file pone.0286259.s006.docx]

**S4 Table. Correlation between SARS-CoV-2 concentrations in two schools and wastewater treatment plant (WWTP)**

| **“A3-2-Primary” leads WWTP data** | | | | | |
| --- | --- | --- | --- | --- | --- |
| Lag -3 week | | **Lag -2 week** | | Lag -1 week | |
| Pearson (r) | P-value | Pearson (r) | P-value | Pearson (r) | P-value |
| 0.46 | 0.001 | **0.51** | **0.0003** | 0.19 | 0.2 |
| **“A3-3-Primary” leads WWTP data** | | | | | |
| Lag -3 week | | **Lag -2 week** | | Lag -1 week | |
| Pearson (r) | P-value | Pearson (r) | P-value | Pearson (r) | P-value |
| 0.36 | 0.02 | **0.40** | **0.01** | 0.14 | 0.30 |
