# Supplementary material for: Wastewater monitoring for detection of public health markers during the COVID-19 pandemic: Near-source monitoring of schools in England over an academic year
Source: PLoS One. 2023 May 30;18(5):e0286259. doi: 10.1371/journal.pone.0286259 (PMC10228768; doi:10.1371/journal.pone.0286259)
Supplement: S5 Table — (DOCX) [file pone.0286259.s007.docx]

**S5 Table. Kitchen spaces and kitchen usage in schools as potential contributors of ammonia and phosphate levels in wastewater**

| School | Student number | Kitchen on site | Lunch preparation on site | Lunch prepared offsite |
| --- | --- | --- | --- | --- |
| A1-1-Secondary | 908 | Y | Y | N |
| A1-2-Primary | 624 | Y | Y | N |
| A1-3-Secondary (A/B) | 2061 | Y | Y | N |
| A2-1-Primary | 335 | Y | Y | N |
| A2-2-Secondary | 497 | Y | Y | N |
| A2-3-Post 16 | N/A | Y | Y | N |
| A2-4-Primary | 419 | Y | Y | N |
| A2-5-Secondary | 1067 | Y | Y | N |
| A2-6-Secondary | 617 | Y | Y | N |
| A3-1-Primary | 615 | Y | Y | N |
| A3-2-Primary | 187 | Y | Y | N |
| A3-3-Primary | 420 | Y | Y | N |
| A3-4-Primary | 199 | Y | Y | N |
| A4-1-Primary | 210 | Y | Y | N |
| A4-2-Primary | 143 | Y | N | Y |
| A4-3-Primary | 183 | Y | N | Y |
| Statistics | | | | |
|  | NH4-N | | PO4-P | |
| Welch’s T-test  (on site vs offsite lunch prep) | t= 1.2944  p= 0.21 | | t= -0.9881  p= 0.33 | |
| Correlation  (Student number) | r= -0.216  p= 0.439 | | r= -0.396  p= 0.143 | |

Y-yes, N-no
