# Supplementary material for: Wastewater monitoring for detection of public health markers during the COVID-19 pandemic: Near-source monitoring of schools in England over an academic year
Source: PLoS One. 2023 May 30;18(5):e0286259. doi: 10.1371/journal.pone.0286259 (PMC10228768; doi:10.1371/journal.pone.0286259)
Supplement: S6 Table — (DOCX) [file pone.0286259.s008.docx]

**S6 Table. Logistic regression of SARS-CoV-2 amplicon level in wastewater and Variant identification**

|  | Estimate | Std.Error | z value | Pr (>\|z\|) |
| --- | --- | --- | --- | --- |
| GC_per_ml_WW_N1 | 7.730e-03 | 3.089e-03 | 2.502 | 0.0123* |
| GC_per_ml_WW_E | 3.225e-04 | 8.267e-05 | 3.902 | 9.56e-05*** |

Significance codes: 0`***` 0.001`**` 0.01`*` 0.05`.` 0.1`` 1

Exponential of coefficients (Estimate) gives ODDs.
