# Supplementary material for: Wastewater monitoring for detection of public health markers during the COVID-19 pandemic: Near-source monitoring of schools in England over an academic year
Source: PLoS One. 2023 May 30;18(5):e0286259. doi: 10.1371/journal.pone.0286259 (PMC10228768; doi:10.1371/journal.pone.0286259)
Supplement: S7 Table — (DOCX) [file pone.0286259.s009.docx]

**S7 Table. Multinomial logistic regression, relationship between variant detection and gene copy of N1 and E concentrations**

|  | Estimate1 | Std.Error1 | Pv-1 | Estimate2 | Std.Error2 | Pv-2 |
| --- | --- | --- | --- | --- | --- | --- |
| GC_per_ml_WW_N1 | 0.007064 | 0.0031883 | 0.027 | 0.0082243 | 0.0031431 | 0.009 |
| GC_per_ml_WW_E | 0.000298 | 0.0001101 | 0.007 | 0.0003437 | 0.0001124 | 0.002 |

Residual Deviance: 123.5132

AIC: 135.5132

Pv < 0.05 is significant
