# Supplementary material for: Wastewater monitoring for detection of public health markers during the COVID-19 pandemic: Near-source monitoring of schools in England over an academic year
Source: PLoS One. 2023 May 30;18(5):e0286259. doi: 10.1371/journal.pone.0286259 (PMC10228768; doi:10.1371/journal.pone.0286259)
Supplement: S1 Data — (PDF) [file pone.0286259.s010.pdf]

Date	CtN1	GC/ml-N1	CtE	GC/ml-E	pH	Conductivity	Dissolved-Oxygen	Total-Suspended-Solids	NH4	PO4	tCOD	sCOD
20/10/2020	37.20	5.60	40.14	1.20	7.40	811.00	6.53	16.00	24.50	1.60	294.00	76.00
20/10/2020					7.56	567.00	9.64	46.00	4.40	2.30	342.00	70.00
20/10/2020					7.53	1097.00	5.10	687.00	57.80	4.10	NA	NA
22/10/2020					7.74	444.00	9.96	4.00	2.60	0.60	489.00	428.00
23/10/2020					8.59	1225.00	3.45	258.00	88.30	2.50	515.00	355.00
04/11/2020					8.25	734.00	9.08	31.00	36.80	2.50	272.00	45.00
04/11/2020	42.57	0.13	39.65	0.27	8.43	980.00	8.06	84.00	56.50	3.50	35.00	<25
04/11/2020	41.91	0.13			6.91	307.00	8.41	514.00	3.70	3.20	148.00	53.00
04/11/2020					7.30	726.00	7.52	201.00	28.40	3.80	304.00	80.00
04/11/2020	35.62	8.67	39.19	0.27	8.36	854.00	5.11	76.00	48.20	2.50	274.00	64.00
04/11/2020			40.24	0.13	6.74	249.00	7.54	79.00	13.50	2.00	375.00	90.00
04/11/2020					7.12	608.00	6.41	62.00	5.00	1.90	746.00	363.00
04/11/2020			35.14	3.73	6.54	57.00	11.81	3.00	1.50	20.90	61.00	<25
09/11/2020					7.65	1156.00	1.34	321.00	>80	20.90	766.00	330.00
09/11/2020	41.68	0.13			8.05	744.00	7.23	98.00	31.30	1.90	167.00	49.00
09/11/2020					7.63	366.00	1.92	146.00	36.10	3.80	221.00	146.00
09/11/2020			43.66	0.00	7.97	800.00	3.32	263.00	36.20	3.20	264.00	193.00
09/11/2020	38.41	1.47	36.63	1.47	7.02	252.00	8.31	67.00	6.40	2.50	93.00	<25
09/11/2020					7.29	320.00	0.70	898.00	22.80	10.60	1500.00	598.00
09/11/2020					8.63	759.00	2.78	280.00	32.10	2.50	364.00	242.00
09/11/2020	38.47	1.33	38.57	0.40	7.29	320.00	0.70	898.00	22.80	10.60	1500.00	598.00
09/11/2020					7.77	325.00	4.02	278.00	13.60	3.50	549.00	285.00
09/11/2020					7.52	531.00	9.78	26.00	2.20	2.00	<25	<25
11/11/2020	37.86	12.00	35.42	21.73	6.14	451.00	6.39	758.00	33.40	17.80	1578.00	351.00
11/11/2020	41.24	1.47			7.40	463.00	8.70	27.00	2.10	2.10	<25	<25
11/11/2020					9.24	1685.00	1.03	112.00	>80	22.90	432.00	255.00
11/11/2020	40.41	2.53	37.27	7.47	7.07	521.00	6.64	42.00	24.40	3.60	<25	<25
11/11/2020					8.62	777.00	2.23	307.00	54.10	2.10	352.00	<25

11/11/2020					9.79	1785.00	2.58	1760.00	>80	19.50	1783.00	588.00
11/11/2020	40.28	2.80			7.17	589.00	6.78	100.00	14.70	1.40	33.00	<25
11/11/2020	42.53	0.93	38.70	3.07	7.16	364.00	3.33	331.00	21.90	4.50	392.00	<25
11/11/2020					6.18	374.00	0.72	81.00	19.80	1.80	198.00	<25
11/11/2020					8.14	795.00	0.90	102.00	48.90	4.20	142.00	<25
16/11/2020					7.67	1069.00	10.95	12.00	5.50	1.10	<25	44.00
16/11/2020	42.97	0.53			8.62	1650.00	8.67	224.00	25.50	<0.5	316.00	35.00
16/11/2020					9.45	3776.00	1.18	223.00	>80	13.40	913.00	207.00
16/11/2020					6.82	2082.00	0.84	1560.00	77.80	57.10	1786.00	757.00
16/11/2020					6.80	703.00	2.38	132.00	0.70	<0.5	239.00	<25
16/11/2020					6.96	1505.00	0.62	1820.00	51.90	46.30	1822.00	363.00
16/11/2020					6.84	655.00	2.47	36.00	3.50	0.80	102.00	10.00
16/11/2020					6.65	638.00	2.53	301.00	1.30	1.80	531.00	71.00
16/11/2020					7.66	1388.00	9.15	52.00	<4.0	0.80	56.00	<25
18/11/2020					8.31	1950.00	5.02	128.00	25.10	3.90	254.00	23.00
18/11/2020	39.18	5.20	37.05	8.40	8.83	1726.00	4.31	56.00	45.10	2.70	84.00	<25
18/11/2020					7.56	3555.00	0.42	2440.00	>80	72.50	2435.00	682.00
18/11/2020					9.17	2098.00	4.26	33.00	47.10	1.60	191.00	36.00
18/11/2020	42.71	0.53			9.12	1976.00	5.18	104.00	40.80	<0.5	190.00	42.00
18/11/2020	43.93	0.27			9.06	959.00	9.42	10.00	39.30	2.90	<25	<25
18/11/2020					8.67	1749.00	1.25	90.00	56.10	4.00	156.00	36.00
18/11/2020	43.76	0.27			6.18	2317.00	1.02	3180.00	99.20	72.50	2960.00	1547.00
18/11/2020					8.02	1585.00	7.25	18.00	17.90	<0.5	47.00	<25
18/11/2020	43.13	0.40			6.85	2067.00	1.55	214.00	41.50	5.40	835.00	364.00
18/11/2020					7.48	956.00	5.64	2999.00	13.80	2.30	151.00	<25
18/11/2020	43.78	0.27			6.35	817.00	1.10	5800.00	12.60	1.60	370.00	108.00
18/11/2020	43.16	0.40			7.90	1610.00	8.83	142.00	6.41	<0.5	<25	<25
16/11/2020					7.45	1798.00	1.84	65.00	33.70	0.80	283.00	167.00
16/11/2020					9.11	2470.00	2.44	119.00	>80	4.60	172.00	<25
16/11/2020					9.51	3497.00	1.17	182.00	>80	4.20	275.00	67.00

23/11/2020	42.87	0.80			7.27	1477.00	6.62	35.00	6.80	13.50	48.00	57.00
23/11/2020	45.59	0.13			8.33	2309.00	2.65	35.00	75.50	7.90	257.00	65.00
23/11/2020					8.99	2391.00	0.66	202.00	110.10	11.30	594.00	198.00
23/11/2020	42.76	0.93			7.74	2281.00	0.06	1076.00	46.00	10.80	1753.00	291.00
23/11/2020	42.31	1.20			7.75	2191.00	0.44	92.00	60.00	5.40	262.00	101.00
23/11/2020	44.09	0.40	39.72	3.07	9.08	3607.00	3.07	1432.00	> 80	6.10	1735.00	232.00
23/11/2020					7.68	1175.00	8.41	10.00	<4.0	0.40	96.00	121.00
23/11/2020	42.35	1.20			6.83	863.00	1.50	77.00	16.80	3.00	237.00	90.00
23/11/2020					8.33	1997.00	0.38	1140.00	37.30	9.90	1035.00	34.00
23/11/2020					7.99	2411.00	0.81	33.00	> 80	9.10	180.00	128.00
23/11/2020					6.90	826.00	0.53	509.00	7.80	4.40	1003.00	104.00
23/11/2020					8.50	3637.00	0.75	2060.00	> 80	22.60	2290.00	609.00
23/11/2020	42.68	0.93			8.33	2309.00	2.65	99.00	75.50	7.90	257.00	135.00
23/11/2020	41.93	1.47			7.49	1558.00	3.93	284.00	15.30	3.60	450.00	116.00
25/11/2020					7.11	1911.00	0.26	558.00	30.60	4.70	1042.00	232.00
25/11/2020					8.89	2568.00	4.93	588.00	93.40	5.20	988.00	93.00
25/11/2020					NA	NA	NA	NA	NA	NA	NA	NA
25/11/2020			38.28	7.47	8.08	1953.00	1.12	597.00	40.40	5.40	754.00	78.00
25/11/2020					7.46	1510.00	0.60	2728.00	11.20	8.70	1907.00	320.00
25/11/2020	44.64	0.27			8.74	1860.00	3.06	164.00	20.90	1.70	335.00	63.00
25/11/2020	43.02	0.80	40.51	1.87	8.12	2038.00	5.02	58.00	24.00	1.80	165.00	68.00
25/11/2020	43.59	0.53			8.63	2171.00	1.85	461.00	43.10	3.20	589.00	57.00
25/11/2020			41.33	1.20	7.99	605.00	5.84	181.00	13.30	2.70	347.00	31.00
25/11/2020					6.68	2695.00	0.44	7760.00	< 80	< 25	2765.00	1692.00
25/11/2020	42.21	1.33	41.57	0.93	8.08	1770.00	1.30	77.00	47.00	4.80	244.00	103.00
25/11/2020					8.48	1935.00	1.61	3940.00	77.10	10.60	1921.00	213.00
25/11/2020			41.91	1.33	7.40	497.00	8.07	22.00	5.70	1.60	44.00	44.00
25/11/2020			35.63	39.60	9.39	2814.00	0.95	46.00	< 80	10.30	238.00	135.00
30/11/2020	37.96	35.47	34.61	24.80	8.51	1103.00	1.33	75.00	71.60	3.10	110.00	39.00
30/11/2020	39.82	12.27	36.13	9.47	7.82	936.00	0.64	964.00	54.70	4.60	1670.00	62.00

30/11/2020	43.56	1.20			7.27	499.00	3.64	77.00	1.70	2.10	128.00	12.00
30/11/2020	36.13	107.60	32.57	86.00	8.81	850.00	1.07	178.00	94.40	6.70	285.00	108.00
30/11/2020	42.00	3.33			6.35	1045.00	0.47	7528.00	85.10	> 25	3512.00	1144.00
30/11/2020	35.17	191.73	31.98	126.13	9.21	1397.00	2.68	207.00	> 80	8.60	405.00	74.00
30/11/2020	41.57	4.00			6.33	143.00	4.14	111.00	3.60	0.60	117.00	5.00
30/11/2020	36.86	69.20	33.13	63.87	7.22	251.00	2.84	88.00	20.30	2.00	142.00	13.00
30/11/2020	41.99	3.07			6.96	719.00	0.22	6240.00	75.10	13.00	3380.00	151.00
30/11/2020					8.60	765.00	2.59	96.00	27.30	1.80	167.00	44.00
01/12/2020	41.47	4.27			9.17	1427.00	1.57	2142.00	> 80	6.00	1801.00	176.00
01/12/2020			35.00	19.07	7.45	583.00	4.10	34.00	18.10	3.40	191.00	62.00
01/12/2020	38.81	23.20	35.34	15.07	6.65	736.00	0.77	517.00	34.50	3.50	875.00	142.00
01/12/2020	41.20	5.07			8.91	1304.00	1.14	888.00	< 80	6.80	1036.00	111.00
01/12/2020	36.02	114.53	32.89	70.40	NA	NA	NA	NA	NA	NA	NA	NA
01/12/2020	38.11	32.67	36.79	6.00	NA	NA	NA	NA	NA	NA	NA	NA
01/12/2020					9.26	784.00	1.14	78.00	> 80	8.90	290.00	66.00
01/12/2020	35.64	143.47	31.87	132.93	7.07	383.00	1.59	85.00	22.60	2.90	124.00	21.00
01/12/2020	40.39	8.27			8.68	860.00	3.81	408.00	> 80	9.60	815.00	93.00
01/12/2020			35.93	11.47	7.29	278.00	7.43	504.00	7.40	3.10	226.00	6.00
01/12/2020	40.90	6.67	36.68	6.40	8.70	773.00	0.62	1980.00	113.90	15.90	1732.00	183.00
01/12/2020					9.17	1427.00	1.57	2142.00	> 80	6.00	1801.00	176.00
01/12/2020	38.69	22.93	35.00	19.07	7.45	583.00	4.10	34.00	18.10	3.40	191.00	62.00
01/12/2020			35.34	15.07	6.65	736.00	0.77	517.00	34.50	3.50	875.00	142.00
01/12/2020					8.91	1304.00	1.14	888.00	< 80	6.80	1036.00	111.00
02/12/2020	41.61	4.00			7.68	670.00	2.22	1184.00	25.90	4.20	2374.00	125.00
02/12/2020	37.18	57.47	32.68	81.20	7.81	688.00	4.42	57.00	30.60	2.60	204.00	45.00
02/12/2020	40.57	7.33			7.67	648.00	1.98	328.00	25.40	3.50	101.00	59.00
02/12/2020					8.43	680.00	5.73	226.00	50.00	5.20	564.00	81.00
02/12/2020					8.94	930.00	2.53	569.00	> 80	5.80	822.00	149.00
02/12/2020	35.02	251.47	30.70	279.20	6.89	562.00	0.94	150.00	22.30	3.20	295.00	178.00
02/12/2020					8.97	1070.00	2.29	563.00	> 80	5.20	869.00	117.00

03/12/2020					NA	NA	NA	NA	NA	NA	NA	NA
03/12/2020	42.19	2.80			NA	NA	NA	NA	NA	NA	NA	NA
03/12/2020					NA	NA	NA	NA	NA	NA	NA	NA
03/12/2020	34.35	314.13	31.51	306.67	NA	NA	NA	NA	NA	NA	NA	NA
03/12/2020	43.28	2.93			NA	NA	NA	NA	NA	NA	NA	NA
03/12/2020	42.50	4.53	36.30	4.53	NA	NA	NA	NA	NA	NA	NA	NA
03/12/2020	41.27	9.07			NA	NA	NA	NA	NA	NA	NA	NA
03/12/2020	41.62	8.27	35.47	8.00	NA	NA	NA	NA	NA	NA	NA	NA
03/12/2020	41.24	11.07	36.19	4.80	NA	NA	NA	NA	NA	NA	NA	NA
03/12/2020	42.91	3.60			NA	NA	NA	NA	NA	NA	NA	NA
03/12/2020	42.22	6.00	35.27	9.20	NA	NA	NA	NA	NA	NA	NA	NA
03/12/2020	41.15	9.73	36.34	4.40	NA	NA	NA	NA	NA	NA	NA	NA
07/12/2020	43.57	2.53			NA	NA	NA	NA	NA	NA	NA	NA
07/12/2020	42.58	4.53	38.84	0.80	NA	NA	NA	NA	NA	NA	NA	NA
07/12/2020	32.10	1683.87	28.18	1313.07	NA	NA	NA	NA	NA	NA	NA	NA
07/12/2020	42.31	5.07			NA	NA	NA	NA	NA	NA	NA	NA
07/12/2020	40.35	15.33			NA	NA	NA	NA	NA	NA	NA	NA
07/12/2020	43.15	3.07			NA	NA	NA	NA	NA	NA	NA	NA
07/12/2020	41.77	6.93	35.60	7.33	NA	NA	NA	NA	NA	NA	NA	NA
08/12/2020	41.33	10.27			7.84	1997.00	1.51	60.00	32.80	3.50	65.00	31.00
08/12/2020	40.71	12.93			7.44	2426.00	0.49	2052.00	49.90	10.20	447.00	185.00
08/12/2020	41.90	6.40			7.07	5086.00	0.17	9844.00	> 80	25.60	1873.00	1268.00
08/12/2020					7.13	1809.00	1.76	426.00	29.10	3.50	188.00	47.00
08/12/2020					8.96	2533.00	5.77	107.00	90.10	4.30	171.00	38.00
08/12/2020					8.85	2838.00	2.89	529.00	> 80	6.20	821.00	87.00
08/12/2020	36.90	110.27	32.35	73.07	6.52	2475.00	0.17	1496.00	57.80	12.70	449.00	586.00
08/12/2020	42.61	4.27	36.07	5.20	8.79	3492.00	0.41	680.00	> 80	5.40	808.00	149.00
09/12/2020	42.65	2.67	36.54	38.67	9.17	3068.00	1.21	129.00	> 80	11.10	122.00	62.00
09/12/2020					8.25	1222.00	4.08	19.00	44.70	4.00	70.00	24.00
09/12/2020	34.22	559.33			6.98	2094.00	0.25	368.00	48.60	5.70	663.00	56.00

09/12/2020	42.54	2.80	38.23	8.13	9.15	2838.00	0.55	85.00	113.80	3.20	85.00	73.00
09/12/2020	44.64	0.80			9.10	3069.00	0.73	467.00	> 80	4.00	740.00	200.00
09/12/2020	39.98	14.53	34.28	55.20	6.47	2713.00	0.31	3264.00	68.30	15.60	1786.00	923.00
09/12/2020					8.91	3938.00	0.54	348.00	> 80	5.40	429.00	111.00
09/12/2020	42.43	4.67			7.35	2170.00	4.22	207.00	32.50	1.20	283.00	64.00
10/12/2020	40.83	2.13			7.73	2715.00	3.03	0.94	59.20	2.70	363.00	56.00
10/12/2020					7.48	2443.00	2.34	0.78	44.30	2.30	166.00	57.00
10/12/2020					7.59	2419.00	0.36	3.59	51.50	5.60	879.00	84.00
10/12/2020	40.47	2.67			8.68	4054.00	0.28	5.78	> 80	11.10	1426.00	168.00
10/12/2020					7.84	1358.00	9.40	0.06	2.70	1.90	13.00	6.00
10/12/2020					9.45	5734.00	1.56	0.79	> 80	23.30	194.00	85.00
10/12/2020	40.77	2.27			NA	NA	NA	NA	NA	NA	NA	NA
10/12/2020	40.86	4.00			8.55	2576.00	8.54	63.00	90.20	4.80	64.00	39.00
10/12/2020					7.13	1824.00	3.07	274.00	34.90	6.10	306.00	57.00
10/12/2020					7.73	2732.00	7.41	173.00	88.10	5.70	321.00	56.00
10/12/2020	35.46	90.13	36.05	113.87	7.37	3377.00	1.05	2496.00	> 80	25.20	2875.00	664.00
10/12/2020					8.70	4759.00	2.04	571.00	> 80	10.80	597.00	92.00
10/12/2020	39.77	4.53			7.72	2260.00	1.14	10.43	38.50	6.70	1256.00	67.00
02/12/2020			35.07	17.87	7.41	297.00	7.74	421.00	9.20	3.20	529.00	28.00
02/12/2020	41.85	4.13			8.88	988.00	3.34	508.00	> 80	14.40	1013.00	272.00
02/12/2020					9.38	1505.00	1.63	73.00	> 80	17.40	285.00	155.00
02/12/2020	37.19	64.27	32.65	81.87	9.23	1263.00	1.91	1596.00	> 80	10.30	1866.00	187.00
02/12/2020	41.73	3.60	35.70	12.00	6.71	467.00	1.55	203.00	37.00	2.60	422.00	169.00
02/12/2020	40.17	10.13	35.69	12.93	6.73	288.00	2.23	40.00	20.10	1.30	190.00	56.00
14/12/2020					8.22	1126.00	10.36	11.00	4.60	0.90	21.00	20.00
14/12/2020	40.85	2.13			7.62	1127.00	10.33	16.00	4.50	0.80	23.00	20.00
14/12/2020	40.91	2.00			8.63	3673.00	2.09	97.00	> 80	11.60	187.00	106.00
14/12/2020					7.03	5171.00	0.95	1131.00	> 80	13.80	1802.00	651.00
14/12/2020	40.67	2.40	39.80	14.13	8.04	2015.00	7.39	54.00	29.90	3.20	109.00	57.00
14/12/2020	39.39	5.47	41.41	6.00	7.91	3636.00	1.51	300.00	> 80	5.80	844.00	116.00

14/12/2020	40.99	1.87			7.28	1689.00	4.14	212.00	69.40	13.30	493.00	149.00
14/12/2020					7.20	1826.00	1.28	11.00	21.60	1.30	366.00	85.00
18/11/2020					8.83	1726.00	4.31	56.00	45.10	2.70	84.00	<25
18/11/2020					9.17	2098.00	4.26	33.00	47.10	1.60	191.00	36.00
18/11/2020					6.85	2067.00	1.55	214.00	41.50	5.40	835.00	364.00
23/11/2020					7.27	1477.00	6.62	35.00	6.80	13.50	48.00	57.00
23/11/2020					9.08	3607.00	3.07	1432.00	> 80	6.10	1735.00	232.00
23/11/2020					7.68	1175.00	8.41	10.00	<4.0	0.40	96.00	121.00
23/11/2020					8.33	2309.00	2.65	99.00	75.50	7.90	257.00	135.00
25/11/2020					8.08	1953.00	1.12	597.00	40.40	5.40	754.00	78.00
25/11/2020					8.89	2568.00	4.93	588.00	93.40	5.20	988.00	93.00
25/11/2020					7.11	1911.00	0.26	558.00	30.60	4.70	1042.00	232.00
25/11/2020					NA	NA	NA	NA	NA	NA	NA	NA
14/12/2020	38.33	11.33	37.26	58.00	6.45	2288.00	2.06	1084.00	58.10	11.40	1885.00	313.00
14/12/2020			40.57	9.20	8.41	2899.00	7.92	127.00	105.20	6.40	118.00	80.00
14/12/2020	41.16	1.73			8.48	2999.00	2.05	68.00	> 80	11.70	149.00	92.00
14/12/2020	44.47	0.27			7.75	3076.00	5.20	456.00	> 80	11.70	655.00	77.00
14/12/2020					8.37	2052.00	8.77	244.00	43.70	3.50	31.00	30.00
14/12/2020	43.55	0.40			7.47	3355.00	3.40	772.00	> 80	9.80	1150.00	119.00
14/12/2020	38.49	9.47	40.96	7.47	7.98	1977.00	9.09	13.00	47.70	4.90	40.00	36.00
14/12/2020					9.04	3142.00	1.53	206.00	> 80	7.80	400.00	109.00
14/12/2020			39.73	14.67	8.45	3702.00	4.54	150.00	> 80	10.60	218.00	94.00
14/12/2020	40.81	2.13			6.38	588.00	8.03	64.00	7.30	1.10	139.00	47.00
14/12/2020					8.09	3276.00	0.73	52.00	2.50	0.50	73.00	29.00
14/12/2020	40.79	2.13			6.64	701.00	5.38	123.00	8.30	3.00	241.00	72.00
14/12/2020	41.01	1.87	41.90	4.53	7.12	882.00	3.62	106.00	19.20	1.80	215.00	82.00
14/12/2020	40.37	3.07			6.81	886.00	1.39	133.00	17.80	1.90	228.00	70.00
10/12/2020	39.82	4.00			NA	NA	NA	NA	NA	NA	NA	NA
10/12/2020					9.23	3408.00	2.48	0.78	> 80	9.90	134.00	37.00
10/12/2020					9.15	3548.00	0.88	0.82	> 80	9.80	153.00	38.00

10/12/2020					7.05	1667.00	0.80	5.62	75.60	15.80	1062.00	174.00
10/12/2020	38.09	12.27			7.69	1697.00	0.42	0.73	45.40	3.10	204.00	107.00
10/12/2020	41.33	1.60			6.84	780.00	7.26	0.86	6.40	2.40	351.00	42.00
10/12/2020	41.03	1.87			7.38	1404.00	5.47	1.54	28.30	4.00	340.00	73.00
10/12/2020	39.83	4.00	40.11	4.13	7.14	1430.00	5.98	0.69	32.40	4.50	129.00	56.00
10/12/2020					8.90	2603.00	1.04	6.83	> 80	11.30	635.00	55.00
09/12/2020					7.69	1331.00	5.82	2.00	1.80	1.60	10.00	10.00
09/12/2020					7.09	3455.00	0.38	2236.00	91.20	19.90	2093.00	586.00
09/12/2020	43.61	1.47			7.34	2178.00	2.51	852.00	42.80	6.00	1193.00	169.00
09/12/2020					7.83	2005.00	5.82	26.00	25.70	1.20	45.00	19.00
11/12/2020					NA	NA	NA	NA	NA	NA	NA	NA
07/12/2020	41.04	10.40	36.51	4.00	NA	NA	NA	NA	NA	NA	NA	NA
11/12/2020					NA	NA	NA	NA	NA	NA	NA	NA
09/12/2020					9.11	2161.00	0.22	61.00	97.40	8.40	118.00	62.00
11/12/2020					NA	NA	NA	NA	NA	NA	NA	NA
07/12/2020	41.07	11.07	36.53	3.87	NA	NA	NA	NA	NA	NA	NA	NA
09/12/2020	42.77	2.80	40.42	3.33	6.48	852.00	0.45	147.00	2.90	0.90	109.00	26.00
11/12/2020	41.29	6.00			NA	NA	NA	NA	NA	NA	NA	NA
11/12/2020					NA	NA	NA	NA	NA	NA	NA	NA
08/12/2020	35.07	312.40	30.03	345.47	7.08	2457.00	7.63	17.00	15.30	1.80	51.00	21.00
09/12/2020	41.30	6.40	35.87	20.53	7.35	1911.00	0.83	133.00	46.20	4.50	118.00	56.00
08/12/2020					6.94	735.00	4.57	26.00	15.10	1.50	110.00	43.00
09/12/2020	42.20	3.87			6.76	856.00	0.30	272.00	3.50	1.30	235.00	73.00
08/12/2020			29.77	406.80	6.92	2372.00	5.69	83.00	15.60	1.80	106.00	26.00
09/12/2020	41.06	7.73	45.80	0.13	7.66	1888.00	0.73	68.00	44.40	4.30	47.00	41.00
11/12/2020					NA	NA	NA	NA	NA	NA	NA	NA
08/12/2020	42.77	2.53			8.31	2836.00	2.59	1028.00	> 80	12.70	1140.00	106.00
09/12/2020					9.08	2352.00	0.68	102.00	112.00	8.00	100.00	68.00
11/12/2020					NA	NA	NA	NA	NA	NA	NA	NA
08/12/2020	44.74	1.20			9.30	3582.00	3.41	49.00	> 80	12.60	83.00	63.00

09/12/2020					6.85	4978.00	0.20	528.00	> 80	23.40	1846.00	1008.00
11/12/2020	41.33	5.73	39.60	11.20	NA	NA	NA	NA	NA	NA	NA	NA
08/12/2020	40.60	10.40	36.42	4.13	9.12	2896.00	0.75	614.00	> 80	9.40	424.00	108.00
09/12/2020			45.71	0.13	6.02	4896.00	0.25	298.57	> 80	22.20	891.00	473.00
15/12/2020					8.24	1655.00	10.36	28.00	27.90	3.10	60.00	20.00
15/12/2020	39.88	7.73	39.56	19.07	6.64	1790.00	0.92	1117.00	26.70	3.30	899.00	88.00
15/12/2020					8.58	3674.00	0.69	1392.00	> 80	9.10	1552.00	223.00
15/12/2020	37.41	4.93			6.26	2082.00	0.97	3140.00	35.50	7.10	2713.00	827.00
15/12/2020					8.84	4085.00	0.43	237.00	> 80	5.60	283.00	79.00
15/12/2020					8.92	5052.00	1.35	756.00	> 80	13.70	852.00	169.00
15/12/2020	42.79	2.00			9.07	3240.00	1.00	151.00	> 80	9.30	249.00	85.00
15/12/2020			42.58	3.07	8.08	2827.00	0.67	65.00	97.50	6.70	242.00	119.00
15/12/2020					8.09	3276.00	0.73	254.00	> 80	9.30	337.00	127.00
11/12/2020					NA	NA	NA	NA	NA	NA	NA	NA
11/12/2020	41.34	5.73			NA	NA	NA	NA	NA	NA	NA	NA
15/12/2020	31.03	189.87	33.43	416.67	7.42	1516.00	4.56	5.00	7.50	0.90	NA	30.00
15/12/2020	34.64	190.67	37.37	46.27	7.24	1787.00	3.74	26.00	20.40	1.20	NA	47.00
15/12/2020			38.76	25.20	8.80	3945.00	2.33	380.00	>80	14.70	607.00	121.00
15/12/2020					8.61	4053.00	4.07	439.00	> 80	16.00	678.00	98.00
15/12/2020	36.92	38.27	37.29	87.07	7.26	1664.00	4.12	9.00	37.00	4.10	130.00	44.00
15/12/2020	35.04	112.67	36.01	116.93	6.96	1653.00	0.52	71.00	38.20	4.00	124.00	47.00
15/12/2020	38.22	18.13	39.19	20.27	6.93	965.00	1.85	338.00	2.90	0.90	240.00	86.00
15/12/2020					7.64	2395.00	3.26	61.00	55.40	5.30	133.00	45.00
15/12/2020					8.16	3549.00	7.75	21.00	> 80	4.20	NA	47.00
15/12/2020					7.09	1984.00	0.38	974.00	32.00	11.40	1273.00	247.00
15/12/2020					8.24	1334.00	10.60	5.00	5.30	1.60	NA	31.00
16/12/2020					8.26	586.00	10.57	32.00	1.10	1.50	349.00	256.00
16/12/2020					7.71	844.00	8.53	158.00	11.60	1.80	415.00	34.00
16/12/2020			43.40	1.60	8.35	1271.00	5.46	1328.00	45.90	6.20	1919.00	152.00
16/12/2020	38.71	13.47	39.48	14.27	7.51	1201.00	8.05	869.00	17.40	6.20	1580.00	367.00

16/12/2020					8.95	1139.00	9.57	92.00	38.10	3.20	157.00	54.00
16/12/2020	42.61	1.87	44.09	1.07	9.07	1419.00	7.84	86.00	44.90	6.70	346.00	202.00
16/12/2020					7.80	890.00	9.94	44.00	12.30	7.30	173.00	116.00
16/12/2020					8.75	2156.00	7.58	145.00	66.20	13.50	456.00	304.00
16/12/2020					NA	NA	NA	NA	NA	NA	NA	NA
16/12/2020	36.78	74.13	37.69	35.33	NA	NA	NA	NA	NA	NA	NA	NA
16/12/2020					NA	NA	NA	NA	NA	NA	NA	NA
16/12/2020					NA	NA	NA	NA	NA	NA	NA	NA
16/12/2020					NA	NA	NA	NA	NA	NA	NA	NA
16/12/2020	41.54	5.20			NA	NA	NA	NA	NA	NA	NA	NA
16/12/2020	40.79	3.73			7.58	260.00	10.24	84.00	0.80	1.70	168.00	31.00
16/12/2020	37.01	35.07	37.93	33.87	7.12	133.00	10.42	61.00	< 4.0	0.30	122.00	55.00
16/12/2020	37.04	35.60	37.36	46.53	7.10	127.00	10.69	55.00	< 4.0	0.30	144.00	77.00
16/12/2020	36.40	51.60	37.11	53.47	9.45	3049.00	9.26	246.00	> 80.0	11.10	829.00	324.00
16/12/2020	36.62	44.13	36.43	78.27	9.50	3247.00	9.54	184.00	> 80.0	11.00	596.00	329.00
16/12/2020					7.04	1112.00	9.42	258.00	8.90	1.00	498.00	107.00
16/12/2020					7.18	510.00	8.66	114.00	3.50	0.80	406.00	104.00
16/12/2020					9.27	1548.00	10.06	71.00	59.60	9.00	310.00	281.00
17/12/2020	39.65	7.47			9.19	2807.00	9.02	232.00	98.30	16.20	1390.00	1133.00
17/12/2020					7.84	1657.00	10.05	45.00	9.30	5.20	131.00	79.00
17/12/2020	41.02	3.33			7.71	1513.00	9.73	302.00	14.60	3.00	300.00	19.00
17/12/2020					NA	NA	NA	NA	NA	NA	NA	NA
17/12/2020					NA	NA	NA	NA	NA	NA	NA	NA
17/12/2020	39.67	15.20	39.80	10.00	NA	NA	NA	NA	NA	NA	NA	NA
17/12/2020	38.04	37.60	39.44	12.27	NA	NA	NA	NA	NA	NA	NA	NA
17/12/2020					9.00	2207.00	11.09	626.00	76.20	6.20	1145.00	225.00
17/12/2020					8.21	2344.00	8.75	1176.00	37.00	5.50	1639.00	481.00
17/12/2020	41.02	3.33			9.08	2477.00	10.32	128.00	94.20	5.70	488.00	162.00
17/12/2020	34.75	231.20	36.66	63.47	NA	NA	NA	NA	NA	NA	NA	NA
17/12/2020	33.21	632.93	34.72	212.53	NA	NA	NA	NA	NA	NA	NA	NA

17/12/2020					9.35	2231.00	6.28	884.00	109.30	10.40	1789.00	506.00
17/12/2020	40.75	3.87			9.03	1728.00	2.35	73.00	51.00	4.70	321.00	167.00
17/12/2020	40.61	4.27			8.97	2117.00	3.78	215.00	49.50	6.90	369.00	140.00
17/12/2020					8.84	1168.00	8.12	262.00	27.50	5.20	1074.00	599.00
17/12/2020	37.48	26.93	37.92	34.13	6.99	634.00	7.69	79.00	0.60	1.90	150.00	55.00
17/12/2020	39.14	10.00			7.38	2751.00	9.29	1056.00	23.20	4.60	1354.00	219.00
17/12/2020	39.92	6.27			7.31	2683.00	5.17	1698.00	23.00	4.70	1606.00	277.00
17/12/2020					9.61	2170.00	8.39	417.00	104.90	9.00	842.00	250.00
17/12/2020					9.65	2866.00	12.10	660.00	111.40	8.40	1238.00	270.00
17/12/2020					9.53	2001.00	8.51	445.00	97.80	7.70	1402.00	265.00
17/12/2020					9.61	3265.00	8.65	3118.00	> 80	10.30	1915.00	516.00
17/12/2020					8.42	820.00	10.41	162.00	2.00	3.10	333.00	113.00
17/12/2020					9.41	2274.00	11.01	1246.00	106.60	16.60	2241.00	404.00
12/01/2021					8.22	2566.00	7.95	174.00	83.50	6.60	343.00	123.00
12/01/2021					8.24	2798.00	7.98	1036.00	92.00	7.00	1163.00	97.00
12/01/2021	37.27	5.33	37.06	8.53	7.26	1877.00	7.93	855.00	33.00	2.70	668.00	186.00
13/01/2021					7.50	1351.00		2834.70	106.00			
13/01/2021	37.12	5.87			7.50	1027.00		1448.00	36.90			
13/01/2021					7.50	1029.00		201.30	127.00			
13/01/2021					7.70	1192.00		1581.30	58.00			
13/01/2021					7.80	1183.00		788.00	59.00			
14/01/2021			37.99	4.67	7.80	394.00		1401.30	5.00			
14/01/2021					7.60	535.00		<d.l.	6.00			
14/01/2021					7.70	945.00		<d.l.	43.00			
14/01/2021					7.50	>2000		1348.00	35.00			
14/01/2021	37.00	6.53			7.60	1476.00		19321.30	110.00			
18/01/2021	37.27	5.33	38.07	4.40	7.70	1053.00		1974.70	37.00			
18/01/2021					7.60	876.00		308.00	32.00			
18/01/2021					7.50	1293.00		321.30	93.00			
18/01/2021	38.92	2.13	39.12	2.27	7.60	1806.00		<d.l.	143.00			

18/01/2021					7.70	1067.00		<d.l.	38.00				
18/01/2021					7.10	1104.00		<d.l.	84.00				
19/01/2021	36.38	8.93	36.73	10.67	7.20	835.00		154.70	8.00				
19/01/2021					7.40	633.00		<d.l.	32.00				
19/01/2021					7.40	652.00		368.00	13.00				
19/01/2021	37.46	4.80			7.40	1051.00		<d.l.	62.00				
19/01/2021					7.40	602.00		<d.l.	10.00				
20/01/2021					7.30	755.00		2541.30	<2				
20/01/2021					7.40	540.00		<d.l.	<2				
20/01/2021					7.40	602.00		<d.l.	14.00				
20/01/2021	39.00	3.07			7.40	573.00		848.00	15.00				
20/01/2021					7.40	555.00		<d.l.	21.00				
20/01/2021					7.30	829.00		1054.70	49.00				
21/01/2021					7.30	735.00		<d.l.	7.00				
21/01/2021					7.20	846.00		174.70	28.00				
21/01/2021	38.98	3.07			7.10	>2000		83134.70	142.00				
21/01/2021					7.70	742.00		561.30	45.00				
21/01/2021					7.70	569.00		501.30	8.00				
21/01/2021	38.82	3.33			7.70	575.00		<d.l.	29.00				
25/01/2021					7.50	976.00		<d.l.	22.00				
25/01/2021					6.90	1925.00		73308.00	44.00				
25/01/2021					7.90	1125.00		961.30	55.00				
25/01/2021					8.00	935.00		1921.30	36.00				
25/01/2021	39.10	2.80			8.20	1238.00		428.00	77.00				
25/01/2021					8.00	1719.00		668.00	134.00				
26/01/2021	39.08	2.93			7.50	807.00		2268.00	19.00				
26/01/2021					7.70	1008.00		<d.l.	58.00				
26/01/2021					7.90	1457.00		68.00	105.00				
26/01/2021	39.33	4.40	37.42	0.45	7.80	1066.00		941.30	44.00				
26/01/2021					6.30	>2000		165088.00	104.00				

26/01/2021					6.50	1276.00		<d.l.	49.00			
27/01/2021					7.50	1532.00		1161.30	133.00			
27/01/2021					7.80	708.00		541.30	49.00			
27/01/2021					7.30	1857.00		374.70	128.00			
27/01/2021			35.18	2.32	7.50	687.00		4534.70	32.00			
27/01/2021					7.50	1189.00		<d.l.	96.00			
28/01/2021	35.37	25.60	36.44	1.01	8.00	1464.00		961.30	102.00			
28/01/2021					8.00	1090.00		1761.30	28.00			
28/01/2021					8.70	193.00		794.70	23.00			
28/01/2021			37.32	0.48	8.20	989.00		818.00	21.00			
28/01/2021					8.30	1085.00		678.00	62.00			
NA	36.58	12.53	35.79	2.07				878.00	64.00			
01/02/2021					7.80	1201.00		3628.00	41.00			
01/02/2021					8.20	1885.00		918.00	144.00			
01/02/2021					8.10	808.00		<d.l.	28.00			
01/02/2021	36.65	12.00			7.50	780.00		<d.l.	n.t.			
02/02/2021								<d.l.	15.00			
02/02/2021					8.20	952.00		<d.l.	85.00			
02/02/2021			37.59	0.40	8.20	1210.00		548.00	22.00			
02/02/2021					7.70	1045.00		1628.00	71.00			
02/02/2021					7.60	937.00		3468.00	67.00			
02/02/2021					7.60	1179.00		918.00	23.00			
03/02/2021	35.41	24.80	35.38	2.07	8.00	752.00		688.00	14.00			
03/02/2021	40.02	0.11			8.00	946.00		1668.00	42.00			
03/02/2021					7.70	1096.00		36708.00	121.00			
03/02/2021	37.14	0.77			7.50	1686.00		318.00	96.00			
03/02/2021					7.60	1286.00		778.00	91.00			
03/02/2021	34.79	3.49	36.39	47.39	7.70	1416.00		<d.l.	17.00			
04/02/2021	37.49	0.58			7.80	1034.00		<d.l.	26.00			
04/02/2021	37.85	0.47			7.90	994.00		<d.l.	30.00			

04/02/2021	37.52	0.57			8.00	1012.00		<d.l.	89.00			
04/02/2021					8.00	1317.00		6838.00	74.00			
04/02/2021	39.85	0.13			7.80	1244.00		938.00	22.00			
08/02/2021					7.90	697.00		<d.l.	18.00			
08/02/2021					47.70	1120.00		37081.30	135.00			
08/02/2021					7.50	>2000		1301.30	35.00			
08/02/2021	37.95	0.43			7.80	978.00		2474.70	129.00			
08/02/2021	39.47	0.16			7.60	>2000		3128.00	115.00			
08/02/2021					7.70	1625.00		614.70	82.00			
09/02/2021	37.98	0.42			7.60	1439.00		1688.00	28.00			
09/02/2021					7.60	1013.00		5928.00	110.00			
09/02/2021	37.20	0.70			7.60	1917.00		1174.70	53.00			
09/02/2021					7.80	1186.00		2494.70	49.00			
09/02/2021					7.60	1056.00		3474.70	128.00			
09/02/2021					7.70	1901.00		648.00	104.00			
10/02/2021					8.00	1475.00		1441.30	101.00			
10/02/2021	36.89	0.86			7.40	1489.00		1654.70	36.00			
10/02/2021					7.60	875.00		4514.70	94.00			
10/02/2021	36.26	1.34	38.15	18.08	7.00	>2000		3914.70	151.00			
10/02/2021					8.20	1957.00		314.70	138.00			
11/02/2021					8.40	1677.00		1554.70	81.00			
11/02/2021								3701.30	99.00			
11/02/2021					7.90	>2000		1474.70	92.00			
11/02/2021	37.01	0.81	41.42	10.23	8.10	1447.00		16274.70	n.t.			
11/02/2021								468.00	105.00			
22/02/2021	38.73	0.39	37.36	1.99	7.00	968.00		1368.00	13.00			
22/02/2021	38.59	0.43			7.90	1295.00		2648.00	75.00			
22/02/2021					7.90	1416.00		1214.70	78.00			
22/02/2021	38.88	0.35			8.10	990.00		1354.70	38.00			
22/02/2021					8.10	746.00		1488.00	12.00			

22/02/2021	38.78	0.38			8.10	872.00		1241.30	14.00			
23/02/2021					8.10	1613.00		2074.70	99.00			
23/02/2021	40.38	0.13			7.90	1263.00		1394.70	62.00			
23/02/2021					8.00	997.00		2328.00	29.00			
23/02/2021					7.90	>2000		2321.30	245.00			
23/02/2021					7.30	1632.00		3401.30	95.00			
23/02/2021	39.06	0.31			7.90	928.00		2614.70	20.00			
24/02/2021					8.00	1063.00		2321.30	21.00			
24/02/2021	38.66	0.41			8.00	1332.00		1894.70	65.00			
24/02/2021					7.80	1106.00		<d.l.	44.00			
24/02/2021					7.80	1943.00		<d.l.	130.00			
24/02/2021					7.90	1686.00		654.70	134.00			
24/02/2021			37.00	2.51	7.50	1120.00		<d.l.	26.00			
25/02/2021	38.67	0.41	37.37	1.98	7.50	1103.00		428.00	31.00			
25/02/2021					7.50	1036.00		241.30	15.00			
25/02/2021					7.60	1208.00		1408.00	53.00			
25/02/2021					7.00	1123.00		<d.l.	45.00			
25/02/2021					6.90	1667.00		<d.l.	96.00			
25/02/2021					6.60	1587.00		1281.30	109.00			
01/03/2021					6.70	1346.00		5414.70	33.00			
01/03/2021	41.13	0.02			7.40	1318.00		314.70	76.00			
01/03/2021					6.80	1947.00		<d.l.	81.00			
01/03/2021	41.49	0.00			6.10	1762.00		24068.00	35.00			
01/03/2021					7.40	1823.00		<d.l.	146.00			
01/03/2021	38.04	0.05			7.20	1396.00		<d.l.	85.00			
02/03/2021	38.62	0.03			6.70	1228.00		<d.l.	30.00			
02/03/2021					7.00	1543.00		30794.70	93.00			
02/03/2021					7.50	1328.00		<d.l.	73.00			
02/03/2021			35.65	3.22	7.70	1980.00		<d.l.	142.00			
02/03/2021					7.70	1211.00		<d.l.	44.00			

02/03/2021	36.70	0.12			7.70	1536.00		668.00	96.00			
03/03/2021	38.30	0.04			7.70	1237.00		1928.00	36.00			
03/03/2021			38.72	1.20	8.20	1273.00		<d.l.	70.00			
03/03/2021	38.83	0.14			8.10	1423.00		694.70	111.00			
03/03/2021			42.51	0.09	7.60	1292.00		994.70	62.00			
03/03/2021			43.30	0.05	7.60	1173.00		1234.70	39.00			
03/03/2021	38.55	0.17	43.45	0.05	7.70	1772.00		<d.l.	121.00			
04/03/2021	38.75	0.15			7.60	1123.00		934.70	23.00			
04/03/2021			34.14	29.11	7.70	1052.00		634.70	20.00			
04/03/2021	37.95	0.25	44.51	0.02	7.70	1790.00		1541.30	121.00			
04/03/2021					7.70	1438.00		728.00	86.00			
04/03/2021	37.15	0.43	38.73	1.20	7.30	1233.00		<d.l.	63.00			
04/03/2021	37.96	0.25	39.21	0.86	7.30	1348.00		<d.l.	82.00			
08/03/2021	37.87	0.27			7.30	1164.00		1274.70	41.00			
08/03/2021	38.13	0.23			7.10	708.00		<d.l.	8.00			
08/03/2021	38.46	0.18			7.30	1274.00		408.00	35.00			
08/03/2021			44.24	0.03	7.40	>2000		694.70	180.00			
08/03/2021	37.89	0.27	37.66	2.52	7.30	1480.00		934.70	83.00			
08/03/2021	43.96	0.00	40.50	0.35	7.20	1136.00		1274.70	39.00			
08/03/2021	39.03	0.12			7.20	1106.00		1754.70	51.00			
08/03/2021	37.31	0.39			7.80	1087.00		<d.l.	60.00			
09/03/2021			38.42	1.48	7.50	1027.00		<d.l.	24.00			
09/03/2021			40.39	0.38	7.40	1182.00		<d.l.	29.00			
09/03/2021	38.00	0.25			7.20	974.00		<d.l.	46.00			
09/03/2021					7.20	1185.00		2074.70	50.00			
09/03/2021					7.10	1691.00		61.30	50.00			
09/03/2021					7.70	1078.00		61.30	54.00			
09/03/2021			42.09	0.12	7.80	1329.00		<d.l.	102.00			
09/03/2021	38.02	0.24			7.50	1897.00		2894.70	147.00			
10/03/2021	38.21	0.21			7.10	1169.00		<d.l.	48.00			

10/03/2021	38.11	0.23	44.18	0.03	7.20	1012.00		1141.30	17.00				
10/03/2021	38.40	0.20	37.48	2.85	7.40	1102.00		1121.30	35.00				
10/03/2021	37.61	0.19	38.89	2.41	7.60	1817.00		368.00	152.00				
10/03/2021					7.10	1465.00		1874.70	109.00				
10/03/2021	37.66	0.19			7.40	770.00		454.70	30.00				
10/03/2021	37.16	0.27	43.85	0.11	7.50	937.00		1008.00	38.00				
10/03/2021					7.10	1590.00		1661.30	53.00				
11/03/2021	37.43	0.22	38.51	3.04	7.50	1271.00		1661.30	62.00				
11/03/2021	37.50	0.21			7.50	1076.00		4501.30	38.00				
11/03/2021					7.40	937.00		121.30	22.00				
11/03/2021					7.50	895.00		768.00	32.00				
11/03/2021					7.70	995.00		<d.l.	51.00				
11/03/2021	37.47	0.21			7.80	874.00		<d.l.	38.00				
11/03/2021	37.62	0.19	39.12	2.10	7.50	1178.00		601.30	74.00				
15/03/2021	37.05	0.30	39.16	2.04	7.60	1340.00		668.00	66.00				
15/03/2021					7.70	787.00		1601.30	<5				
15/03/2021					7.60	1195.00		<d.l.	29.00				
15/03/2021	34.98	1.24	35.97	15.02	7.50	>2000		3041.30	148.00				
15/03/2021	38.52	0.20			7.50	1695.00		1614.70	96.00				
15/03/2021	38.08	0.14	42.04	0.34	7.40	1112.00		<d.l.	46.00				
15/03/2021					7.50	1159.00		<d.l.	47.00				
15/03/2021	38.12	0.13			7.50	1185.00		14021.30	85.00				
15/03/2021	37.94	0.15			7.60	1128.00		<d.l.	38.00				
16/03/2021	36.59	0.40			7.70	1342.00		<d.l.	70.00				
16/03/2021					7.80	762.00		<d.l.	<5				
16/03/2021					7.70	1385.00		<d.l.	37.00				
16/03/2021	36.14	0.56	37.76	5.96	7.60	>2000		2961.30	>150				
16/03/2021					7.70	997.00		848.00	34.00				
16/03/2021	37.68	0.18			8.00	1241.00		4361.30	78.00				
16/03/2021					7.70	>2000		4841.30	142.00				

16/03/2021	36.58	0.40			7.70	1287.00		6221.30	59.00			
16/03/2021			44.90	0.06	7.80	938.00		974.70	37.00			
17/03/2021					7.80	1124.00		<d.l.	26.00			
17/03/2021	33.28	4.15	34.56	35.05	7.80	1652.00		<d.l.	114.00			
17/03/2021	37.72	0.18			7.60	715.00		1608.00	2.10			
17/03/2021	37.64	0.19	37.92	4.39	7.60	1217.00		<d.l.	26.20			
17/03/2021	36.79	1.74			7.40	1268.00		168.00	10.80			
17/03/2021					7.50	1544.00		668.00	16.40			
17/03/2021	36.95	1.56			7.60	1582.00		1861.30	25.90			
17/03/2021					7.50	1997.00		1254.70	53.90			
17/03/2021	35.86	3.42	37.29	3.25	7.50	1145.00		<d.l.	21.00			
18/03/2021	36.91	1.60	39.73	3.96	7.20	649.00		261.30	28.40			
18/03/2021					7.70	103.00		2634.70	<5			
18/03/2021					7.50	1264.00		161.30	15.30			
18/03/2021	35.33	5.34	35.59	9.74	7.40	1510.00		<d.l.	27.60			
18/03/2021	37.18	1.31	40.25	0.49	7.30	1189.00		14.70	21.20			
18/03/2021					7.40	493.00		174.70	16.60			
18/03/2021					7.60	1927.00		<d.l.	48.30			
18/03/2021	36.64	1.94			7.40	1508.00		<d.l.	28.60			
18/03/2021					7.70	1492.00		<d.l.	41.70			
22/03/2021	37.25	1.25	37.11	3.65	7.30	1227.00		728.00	20.40			
22/03/2021	37.05	1.44			7.70	146.00		3774.70	<5			
22/03/2021	36.95	2.89	36.98	3.95	7.50	402.00		741.30	14.00			
22/03/2021	35.18	5.95	34.34	22.56	7.50	>2000		<d.l.	111.00			
22/03/2021	37.91	0.77			7.60	1319.00		2034.70	60.90			
22/03/2021					8.10	1434.00		734.70	24.80			
22/03/2021					8.60	420.00		494.70	35.10			
22/03/2021					8.00	1816.00		<d.l.	15.90			
22/03/2021	37.44	1.09			8.00	>2000		2768.00	124.00			
23/03/2021					8.20	1132.00		768.00	13.90			

23/03/2021	37.48	1.06			8.30	724.00		534.70	<5			
23/03/2021	36.69	1.97	35.93	9.31	8.00	1993.00		668.00	59.60			
23/03/2021	37.98	0.73			7.50	1326.00		581.30	22.30			
23/03/2021	37.88	0.79			7.60	1148.00		2014.70	26.00			
23/03/2021					8.40	1431.00		508.00	40.60			
23/03/2021					8.50	251.00		381.30	13.30			
23/03/2021	37.93	0.76			8.00	1825.00		54.70	53.60			
23/03/2021	37.92	0.77	37.21	3.43	7.90	1307.00		1008.00	32.80			
24/03/2021					8.00	1266.00		1441.30	27.00			
24/03/2021	37.25	1.25			8.30	436.00		<d.l.	<5			
24/03/2021					7.90	1862.00		561.30	52.70			
24/03/2021	37.57	0.99	38.22	1.79	7.20	1247.00		<d.l.	16.80			
24/03/2021			36.97	2.27	7.20	986.00		1.30	12.70			
24/03/2021			36.94	2.32	7.10	1260.00		<d.l.	47.70			
24/03/2021	37.36	2.11			7.20	>2000		574.70	32.90			
24/03/2021	40.82	0.16			7.60	288.00		6234.70	14.00			
24/03/2021	37.94	1.30			8.20	>2000		2034.70	60.50			
25/03/2021	36.98	2.63	36.86	2.44	7.90	1176.00		<d.l.	29.10			
25/03/2021					8.00	553.00		1448.00	<5			
25/03/2021	36.88	2.82			7.90	1480.00		<d.l.	52.60			
25/03/2021					8.20	306.00		1014.70	2.90			
25/03/2021					7.40	1699.00		<d.l.	53.00			
25/03/2021					7.50	830.00		<d.l.	30.00			
25/03/2021	40.11	0.27	36.92	2.34	7.40	1332.00		781.30	75.00			
25/03/2021	36.37	4.09	36.95	2.30	7.60	1809.00		368.00	30.00			
25/03/2021			42.05	0.07	8.20	1474.00		6248.00	67.00			
29/03/2021	37.94	1.30			7.10	1393.00		1501.30	113.00			
29/03/2021					7.60	843.00		<d.l.	78.00			
29/03/2021					7.60	1315.00		3868.00	55.00			
29/03/2021					7.60	1843.00		1474.70	110.00			

29/03/2021					7.40	1076.00		<d.l.	47.00			
29/03/2021	37.70	1.55			7.70	>2000		2421.30	130.00			
29/03/2021	37.55	2.02			7.00	1748.00		1114.70	78.00			
29/03/2021					7.20	947.00		648.00	35.00			
29/03/2021					7.00	1433.00		5254.70	77.00			
30/03/2021	35.78	7.55	35.56	6.13	7.40	1304.00		1148.00	64.00			
30/03/2021	37.69	1.56			7.60	1194.00		1828.00	62.00			
30/03/2021					7.40	1137.00		<d.l.	35.00			
30/03/2021	36.99	3.16	38.06	1.07	7.80	1627.00		1101.30	81.00			
30/03/2021	38.53	0.85			7.50	923.00		1074.70	33.00			
30/03/2021	37.77	1.47			7.50	1254.00		<d.l.	65.00			
30/03/2021	38.25	1.03			7.20	1457.00		1121.30	56.00			
30/03/2021					8.40	1875.00		61.30	133.00			
30/03/2021					7.60	>2000		3754.70	149.00			
31/03/2021	37.75	0.57	37.53	1.59	7.40	1528.00		<d.l.	96.00			
31/03/2021					7.40	1034.00		<d.l.	37.00			
31/03/2021					7.70	1434.00		1861.30	42.00			
31/03/2021	42.91	0.02			8.30	1932.00		<d.l.	103.00			
31/03/2021					7.30	955.00		<d.l.	32.00			
31/03/2021	36.90	0.99			7.70	1325.00		748.00	84.00			
31/03/2021					6.70	1647.00		2094.70	59.00			
31/03/2021					8.80	>2000		<d.l.	>150			
31/03/2021					7.80	>2000		2248.00	85.00			
20/04/2021					8.20	1447.00		1.30	149.00			
20/04/2021	39.61	0.53			7.90	1623.00		1301.30	81.00			
20/04/2021	38.04	1.51			8.10	1657.00		1094.70	140.00			
20/04/2021	37.97	1.57			7.00	978.00		1534.70	67.00			
20/04/2021					7.40	1552.00		828.00	65.00			
20/04/2021	38.69	0.98			7.60	>2000		1754.70	141.00			
20/04/2021					7.80	923.00		<d.l.	49.00			

21/04/2021					8.20	>2000		1208.00	124.00			
21/04/2021					7.60	679.00		<d.l.	5.00			
21/04/2021					7.60	1305.00		<d.l.	37.00			
21/04/2021	40.21	0.36			8.40	1514.00		168.00	92.00			
21/04/2021					7.30	939.00		294.70	34.00			
21/04/2021					7.80	1144.00		321.30	88.00			
21/04/2021					7.10	1442.00		54.70	69.00			
22/04/2021					7.30	>2000		3754.70	134.00			
22/04/2021	36.13	5.07			7.80	638.00		1214.70	<5			
22/04/2021					7.60	1615.00		1814.70	63.00			
22/04/2021			39.13	0.43	7.60	1792.00		1621.30	100.00			
22/04/2021	39.82	0.54			7.40	1206.00		588.00	44.00			
22/04/2021					7.40	1144.00		<d.l.	90.00			
22/04/2021	37.33	2.42			7.20	1523.00		6548.00	56.00			
22/04/2021	36.94	3.06			7.10	1099.00		1281.30	43.00			
26/04/2021					7.70	>2000		3608.00	128.00			
26/04/2021					7.80	653.00		4428.00	<5			
26/04/2021					7.70	1462.00		<d.l.	48.00			
26/04/2021					7.80	>2000		1501.30	154.00			
26/04/2021					7.50	1190.00		2094.70	39.00			
26/04/2021					7.40	1478.00		<d.l.	70.00			
26/04/2021					8.40	1133.00		2114.70	62.00			
27/04/2021	38.83	0.95			8.60	1437.00		4514.70	59.00			
27/04/2021	36.91	3.12			7.60	733.00		<d.l.	5.00			
27/04/2021					7.70	1435.00		1168.00	37.00			
27/04/2021					7.80	1837.00		288.00	100.00			
27/04/2021					7.90	1063.00		<d.l.	17.00			
27/04/2021	36.92	3.11			7.20	1528.00		2254.70	74.00			
27/04/2021	36.93	3.08			7.80	1429.00		<d.l.	16.00			
28/04/2021					8.60	1792.00		<d.l.	110.00			

28/04/2021					8.10	947.00		1001.30	<5			
28/04/2021					8.30	1447.00		534.70	18.00			
28/04/2021					7.70	1953.00		148.00	141.00			
28/04/2021	37.13	2.73			6.80	706.00		721.30	8.00			
28/04/2021					8.20	1149.00		274.70	16.00			
28/04/2021	37.41	2.30			7.10	1856.00		2028.00	25.00			
29/04/2021	38.11	0.92			8.40	1542.00		1034.70	18.00			
29/04/2021					7.50	998.00		228.00	<5			
29/04/2021					7.50	1245.00		<d.l.	10.00			
29/04/2021					8.20	1493.00		294.70	41.00			
29/04/2021					6.80	1343.00		914.70	63.00			
29/04/2021					7.30	876.00		3048.00	5.00			
29/04/2021					8.00	1118.00		1834.70	22.00			
04/05/2021					8.80	1387.00		<d.l.	23.00			
04/05/2021					7.90	751.00		<d.l.	<5			
04/05/2021					7.70	847.00		48.00	11.00			
04/05/2021	38.26	0.83			8.10	1320.00		478.00	41.00			
04/05/2021	38.32	0.79			7.40	718.00		358.00	4.00			
04/05/2021	38.33	0.79			7.30	810.00		1138.00	7.00			
04/05/2021	38.24	0.85			7.30	576.00		2128.00	<5			
05/05/2021					8.50	1301.00		<d.l.	49.00			
05/05/2021					7.90	713.00		11268.00	<5			
05/05/2021	39.80	0.29			7.60	1033.00		<d.l.	12.00			
05/05/2021	37.25	1.68			8.60	1206.00		1788.00	49.00			
05/05/2021					7.60	851.00		1728.00	10.00			
05/05/2021					7.30	781.00		168.00	10.00			
05/05/2021					7.50	739.00		548.00	13.00			
06/05/2021	37.10	1.43			8.20	1054.00		<d.l.	32.00			
06/05/2021	36.72	1.85			8.00	775.00		<d.l.	<5			
06/05/2021					7.90	1063.00		1578.00	16.00			

06/05/2021	38.20	0.69			8.20	1176.00		88.00	44.00			
06/05/2021					7.80	798.00		1068.00	11.00			
06/05/2021	35.67	3.74			7.60	721.00		868.00	11.00			
06/05/2021	37.39	1.71			7.50	769.00		<d.l.	7.00			
10/05/2021					8.70	1244.00		<d.l.	28.00			
10/05/2021					8.00	639.00		<d.l.	<5			
10/05/2021					7.90	863.00		<d.l.	10.00			
10/05/2021					7.30	785.00		648.00	7.00			
10/05/2021	40.52	0.37			7.50	897.00		968.00	9.00			
11/05/2021	37.08	1.45			8.50	1304.00		1768.00	35.00			
11/05/2021					8.40	619.00		1048.00	<5			
11/05/2021	36.16	2.69			8.20	1061.00		<d.l.	12.00			
11/05/2021	35.67	3.73			8.30	1682.00		<d.l.	50.00			
11/05/2021	34.58	7.73			7.50	733.00		1308.00	17.00			
11/05/2021					7.30	711.00		2028.00	6.00			
11/05/2021	36.00	2.99			8.60	1253.00		578.00	78.00			
12/05/2021	42.58	0.04			8.70	1272.00		<d.l.	36.00			
12/05/2021					8.60	742.00		868.00	11.00			
12/05/2021	36.47	2.19			8.20	1158.00		<d.l.	18.00			
12/05/2021					8.40	1371.00		<d.l.	75.00			
12/05/2021	40.58	0.14			7.80	811.00		718.00	18.00			
12/05/2021					7.70	771.00		938.00	22.00			
12/05/2021	38.17	1.22			8.60	1153.00		1008.00	81.00			
13/05/2021					8.60	1193.00		<d.l.	35.00			
13/05/2021					8.50	665.00		1178.00	5.00			
13/05/2021	38.09	1.67			8.60	660.00		<d.l.	6.00			
13/05/2021					8.70	1305.00		<d.l.	82.00			
13/05/2021					7.50	551.00		<d.l.	6.00			
13/05/2021					8.50	821.00		1538.00	53.00			
13/05/2021					8.40	815.00		<d.l.	48.00			

17/05/2021			44.57	0.01	8.40	1206.00		788.00	33.00			
17/05/2021	38.07	1.70			8.20	618.00		<d.l.	<5			
17/05/2021	38.07	1.71			8.20	423.00		<d.l.	4.00			
17/05/2021					8.00	1230.00		<d.l.	28.00			
17/05/2021	37.29	2.88			8.60	1165.00		1088.00	67.00			
17/05/2021					8.80	1109.00		1228.00	90.00			
17/05/2021	38.14	1.63			8.10	745.00		648.00	7.00			
18/05/2021					8.80	1316.00		968.00	104.00			
18/05/2021					8.40	568.00		428.00	<5			
18/05/2021					8.30	659.00		898.00	5.00			
18/05/2021					8.80	1442.00		<d.l.	44.00			
18/05/2021					7.20	685.00		<d.l.	18.00			
18/05/2021					8.80	1049.00		108.00	69.00			
19/05/2021	40.76	0.28			8.80	1504.00		78.00	96.00			
19/05/2021					8.10	748.00		1568.00	8.00			
19/05/2021			38.96	0.61	7.90	943.00		<d.l.	15.00			
19/05/2021					9.00	1609.00		4598.00	109.00			
19/05/2021					7.00	1059.00		<d.l.	8.00			
19/05/2021					8.30	1356.00		2838.00	88.00			
19/05/2021	38.01	1.77			8.90	1286.00		348.00	89.00			
20/05/2021					8.50	1383.00		4258.00	69.00			
20/05/2021	38.75	1.87			8.60	874.00		548.00	7.00			
20/05/2021					8.40	1099.00		258.00	35.00			
20/05/2021					8.60	1901.00		1538.00	146.00			
20/05/2021					9.10	1228.00		2108.00	103.00			
20/05/2021					8.90	1380.00		1658.00	142.00			
20/05/2021					8.00	705.00		1198.00	13.00			
24/05/2021	38.54	2.18	38.65	0.50	8.10	1170.00		1698.00	40.00			
24/05/2021	38.46	2.35			7.70	693.00		3298.00	7.00			
24/05/2021					7.50	698.00		518.00	<5			

24/05/2021					8.50	1373.00		348.00	24.00				
24/05/2021					8.80	1491.00		868.00	55.00				
24/05/2021					8.30	621.00		<d.l.	59.00				
24/05/2021					8.20	1222.00		<d.l.	5.00				
25/05/2021					8.30	1345.00		138.00	64.00				
25/05/2021	39.68	0.96			7.90	662.00		1838.00	7.00				
25/05/2021					7.80	793.00		928.00	5.00				
25/05/2021					8.00	1602.00		458.00	31.00				
25/05/2021					8.70	1262.00		11178.00	67.00				
25/05/2021					8.80	1387.00		1478.00	83.00				
25/05/2021					8.20	761.00		998.00	18.00				
26/05/2021	37.95	3.33			7.80	1228.00		298.00	42.00				
26/05/2021					7.60	633.00		1508.00	<5				
26/05/2021	39.04	1.52			7.50	939.00		<d.l.	10.00				
26/05/2021	41.11	0.34			8.60	1720.00		<d.l.	81.00				
26/05/2021	38.46	2.31			8.80	1602.00		1578.00	98.00				
26/05/2021					8.80	1320.00		728.00	72.00				
26/05/2021					8.00	895.00		1568.00	22.00				
27/05/2021					7.60	1796.00		548.00	46.00				
27/05/2021	39.01	1.86			7.70	764.00		1468.00	<5				
27/05/2021					7.50	1082.00		<d.l.	15.00				
27/05/2021					8.90	1894.00		808.00	123.00				
27/05/2021	38.48	2.68			8.90	1522.00		2468.00	82.00				
27/05/2021	42.99	0.13			8.20	1087.00		958.00	38.00				
27/05/2021					8.80	1340.00		998.00	92.00				
08/06/2021	35.25	18.05	34.22	15.88	8.50	1432.00		3074.00	58.00				
08/06/2021					7.70	1030.00		<d.l.	16.00				
08/06/2021	38.40	2.07			7.20	925.00		2537.00	31.00				
08/06/2021					8.50	1134.00		2893.00	67.00				
08/06/2021					9.40	1568.00		92.00	87.00				

09/06/2021	35.18	18.83	35.64	6.42	8.70	1187.00		<d.l.	41.00			
09/06/2021					7.80	1123.00		1837.00	21.00			
09/06/2021					6.70	732.00		<d.l.	10.00			
09/06/2021					8.90	1040.00		1513.00	44.00			
10/06/2021	36.89	7.27	40.76	0.44	8.70	1508.00		880.00	53.00			
10/06/2021					8.60	1200.00		125.00	19.00			
10/06/2021					8.40	920.00		2799.00	5.00			
10/06/2021					6.70	811.00		<d.l.	10.00			
10/06/2021	26.74	9198.32	27.43	1126.02	8.60	872.00		<d.l.	35.00			
10/06/2021	38.32	2.68			8.60	1127.00		<d.l.	52.00			
14/06/2021					8.20	1212.00		627.00	36.00			
14/06/2021					7.80	1172.00		1561.00	15.00			
14/06/2021	37.90	3.60			7.80	729.00		<d.l.	<5			
14/06/2021					6.50	875.00		<d.l.	18.00			
14/06/2021	27.65	4715.42	28.03	744.80	6.80	1816.00		32662.00	96.00			
14/06/2021					8.80	1454.00		2634.00	126.00			
15/06/2021	38.07	3.20	37.38	2.31	8.90	1432.00		172.00	81.00			
15/06/2021					7.90	1210.00		978.00	24.00			
15/06/2021			38.60	1.08	8.00	1638.00		682.00	33.00			
15/06/2021					6.70	749.00		3562.00	11.00			
15/06/2021	29.25	1564.61	29.48	318.19	8.60	1156.00		1348.00	77.00			
15/06/2021	37.84	3.74			9.00	1550.00		3284.00	116.00			
16/06/2021	37.31	3.28			7.90	>2000		1896.00	79.00			
16/06/2021	37.26	3.39			8.00	1655.00		136.00	20.00			
16/06/2021					6.60	920.00		2044.00	14.00			
16/06/2021	29.09	1161.35	29.62	238.26	8.20	1519.00		1889.00	98.00			
17/06/2021	36.31	6.67			8.60	1483.00		2061.00	64.00			
17/06/2021					8.50	988.00		308.00	15.00			
17/06/2021	37.11	3.78			6.60	791.00		1177.00	15.00			
17/06/2021	28.24	2373.36	28.46	613.81	8.40	1363.00		2237.00	64.00			

17/06/2021					9.20	>2000		2586.00	148.00				
21/06/2021													
21/06/2021													
21/06/2021													
21/06/2021	28.75	153.73	30.38	132.00									
21/06/2021	29.94	60.53	31.41	61.73									
21/06/2021													
21/06/2021													
22/06/2021													
22/06/2021													
22/06/2021													
22/06/2021	31.53	17.60	32.52	27.33									
22/06/2021	30.96	27.33	32.29	32.27									
22/06/2021													
22/06/2021													
23/06/2021					8.80	1442.00		1729.00	64.00				
23/06/2021	37.82	2.76			8.70	1175.00		352.00	23.00				
23/06/2021	40.33	0.46			8.80	1712.00		1198.00	102.00				
23/06/2021					7.00	824.00		1726.00	21.00				
23/06/2021	32.83	98.71	34.69	12.46	8.20	1625.00		1541.00	105.00				
23/06/2021	37.09	4.65			8.90	1101.00		179.00	54.00				
24/06/2021					8.40	1141.00		<d.l.	42.00				
24/06/2021					7.90	1125.00		726.00	20.00				
24/06/2021					8.60	1364.00		1514.00	56.00				
24/06/2021					6.70	941.00		708.00	26.00				
24/06/2021	35.41	17.65	37.73	1.69	8.60	1422.00		235.00	81.00				
24/06/2021	37.40	3.73			9.10	1466.00		939.00	100.00				
28/06/2021					7.90	1288.00		<d.l.	36.00				
28/06/2021					8.00	893.00		<d.l.	10.00				
28/06/2021					8.10	1102.00		44.00	35.00				

28/06/2021					6.80	658.00		2040.00	11.00				
28/06/2021	34.14	56.45	34.53	7.39	7.70	1214.00		867.00	60.00				
28/06/2021	36.84	8.05			8.90	554.00		2527.00	21.00				
29/06/2021					7.90	1342.00		<d.l.	31.00				
29/06/2021					8.00	1115.00		<d.l.	19.00				
29/06/2021	37.80	3.10			7.90	1123.00		1111.00	23.00				
29/06/2021					7.20	808.00		399.00	17.00				
29/06/2021	36.58	8.18	37.01	1.92	8.80	1088.00		<d.l.	64.00				
29/06/2021	35.46	19.27	37.21	1.39	8.90	1005.00		508.00	50.00				
30/06/2021	37.07	5.59			8.60	1481.00		269.10	71.00				
30/06/2021					8.30	1319.00		1570.00	22.00				
30/06/2021	37.20	5.06			8.20	1137.00		1686.00	13.00				
30/06/2021					7.20	704.00		842.00	12.00				
30/06/2021	33.13	235.85	36.37	39.47	8.30	1492.00		638.00	115.00				
30/06/2021					8.40	909.00		1704.00	28.00				
01/07/2021	37.29	4.73			8.10	1225.00		2925.00	40.00				
01/07/2021	37.22	4.97			8.00	1206.00		1626.00	20.00				
01/07/2021					8.00	997.00		<d.l.	9.00				
01/07/2021					6.70	743.00		<d.l.	14.00				
01/07/2021	34.85	32.10	35.61	6.23	8.60	1124.00		3694.00	62.00				
01/07/2021					8.80	1407.00		822.00	61.00				
05/07/2021					7.30	1130.00							
05/07/2021					8.40	1100.00							
05/07/2021					8.80	1320.00							
05/07/2021	39.16	0.60	36.33	19.92	8.30	840.00							
05/07/2021	42.16	0.26			8.80	1200.00							
05/07/2021					7.30	540.00							
06/07/2021					7.90	650.00							
06/07/2021					8.20	800.00							
06/07/2021	42.24	0.23			8.50	1070.00							

06/07/2021	38.04	1.35	36.55	16.65	8.10	930.00						
06/07/2021					8.80	1110.00						
06/07/2021					7.20	570.00						
07/07/2021	44.06	0.02			7.60	790.00						
07/07/2021					8.20	1080.00						
07/07/2021					8.60	1000.00						
07/07/2021	39.67	0.41	36.33	19.92	8.00	740.00						
07/07/2021					9.30	1430.00						
07/07/2021					7.50	440.00						
08/07/2021					7.60	970.00						
08/07/2021					9.20	1070.00						
08/07/2021					9.30	1380.00						
08/07/2021	42.30	0.44			7.70	1250.00						
08/07/2021	42.28	0.45			9.20	1200.00						
08/07/2021					7.30	620.00						
12/07/2021					8.00	980.00						
12/07/2021					8.80	1450.00						
12/07/2021			41.68	5.00	8.70	900.00						
12/07/2021					8.40	490.00						
13/07/2021					7.30	780.00						
13/07/2021	42.25	0.47			8.40	1320.00						
13/07/2021					8.50	1020.00						
13/07/2021	41.71	1.00			7.80	960.00						
13/07/2021			41.68	5.00	8.50	720.00						
13/07/2021					7.20	550.00						
14/07/2021			37.85	15.12	7.60	970.00						
14/07/2021					8.50	880.00						
14/07/2021			42.69	1.29	8.80	1230.00						
14/07/2021			35.24	81.77	8.60	1040.00						
14/07/2021					8.10	570.00						

14/07/2021					6.90	580.00						
15/07/2021			37.59	19.30	8.00	970.00						
15/07/2021	42.25	0.48	37.38	19.36	7.60	1020.00						
15/07/2021					8.60	1470.00						
